# Association of Direct-to-Consumer Advertising of Prescription Drugs With Consumer Health-Related Intentions and Beliefs Among Individuals at Risk of Cardiovascular Disease

**DOI:** 10.1001/jamahealthforum.2022.2570

**Published:** 2022-08-12

**Authors:** Matthew D. Eisenberg, Yashaswini Singh, Neeraj Sood

**Affiliations:** 1 Department of Health Policy and Management, Johns Hopkins Bloomberg School of Public Health, Baltimore, Maryland; 2 Sol Price School of Public Policy, University of Southern California, Los Angeles

## Abstract

**Importance:**

Consumers in the US are exposed to unprecedented high levels of direct-to-consumer advertising (DTCA) for prescription drugs, yet there is limited evidence regarding their effect on health-related intentions and beliefs.

**Objective:**

To provide evidence on the association of DTCA for prescription drugs with consumer health-related intentions and beliefs.

**Design, Setting, and Participants:**

This cross-sectional study recruited participants from a nationally representative sample of individuals at high risk of cardiovascular disease. Participants were randomly assigned into 1 of 3 study arms: (1) exposure to DTCA for heart disease medications (treatment 1 [n = 926]), (2) exposure to DTCA for heart disease medications with price disclosure (treatment 2 [n = 921]), (3) and exposure to nonpharmaceutical advertising (control group [n = 902]). Each study arm viewed five 1-minute video advertisements, totaling 5 minutes of advertising exposure. The 2 treatment arms viewed pharmaceutical advertising videos for 4 heart disease medications, and the control arm viewed nonpharmaceutical advertising videos. Participants then completed a survey questionnaire to measure medication- and lifestyle-related intentions and health-related beliefs and perceptions.

**Exposures:**

Direct-to-consumer advertising for heart disease medications (treatment 1), DTCA for heart disease medications with price disclosure (treatment 2), and nonpharmaceutical advertising (control group).

**Main Outcomes and Measures:**

The primary outcomes included ordinal measures of medication- and lifestyle-related intentions, health-related beliefs, and brand perceptions.

**Results:**

Among the 2874 included participants (mean [SD] age, 53.8 [7.1] years; 1549 [54%] male) χ^2^ tests confirmed that there were no statistically significant differences in baseline demographic characteristics across study arms. There was a positive association between DTCA and medication-related behavioral intentions, including intention to switch medication (marginal effect [ME] = 0.004; *P* = .002) and engage in information-seeking behaviors (ME = 0.02; *P* = .01). There was no evidence that pharmaceutical DTCA discouraged use of nonpharmacological lifestyle interventions that can help manage heart disease (eg, diet and exercise), and DTCA exposure also had a positive association with consumers’ favorable perceptions of pharmaceutical manufacturers (competence: ME = 0.03; *P* = .01; innovative: ME = 0.03; *P* = .008). There was no evidence for differential associations of price disclosures in DTCA.

**Conclusions and Relevance:**

In this cross-sectional study, results showed that brief exposure to pharmaceutical DTCA had a large and positive association with medication-related demand intentions with no offsetting negative spillovers on lifestyle-related intentions. Lack of associations with price disclosure in DTCA suggests that policy makers should consider alternative strategies to promote value-based decision-making for prescription drugs.

## Key Points

**Question:**

What is the association of direct-to-consumer advertising of prescription drugs with consumer intentions and beliefs related to prescription drugs and health-related behaviors?

**Findings:**

In this cross-sectional study of 2874 individuals at risk of cardiovascular disease, participants in the treatment groups viewed 5 minutes of prescription drug advertisements while those in the control group viewed advertisements for other consumer products. Participants in the treatment groups were more likely to report favorable perceptions of pharmaceutical manufacturers and medication-related behavior intentions, such as switching medications, though there were no statistically significant differences in behavior intentions for diet and exercise between the treatment and control groups.

**Meaning:**

Brief exposure to prescription drug advertisements has a large and positive association with medication-related demand intentions with no offsetting negative spillovers on lifestyle-related intentions.

## Teaser

This cross-sectional study provides evidence on the association of direct-to-consumer-advertising of prescription drugs with consumer health-related intentions and beliefs among individuals at risk of cardiovascular disease.

## Introduction

Consumers in the US are exposed to unprecedented levels of pharmaceutical direct-to-consumer advertising (DTCA), with more than $18 billion spent in 2016 through 2018.^1^ Proponents argue that DTCA provides information to consumers, promotes medication adherence, and encourages healthy behaviors. Critics argue that DTCA leads consumers to choose brand-name drugs over lower-cost generics, discourages use of nonpharmacologic treatments, and promotes inappropriate drug prescribing behavior. While DTCA has the potential to affect consumer behavior, the causal link between advertising and behavior change contains many steps. Prior work has identified associations between DTCA and rising prescription drug utilization using observational data^2,3,4,5,6^ and experimental settings.^7^ Patient preferences play only one role in the process because prescribers have a considerable degree of influence to direct, modify, or nullify the effect of DTCA on consumer behavior, which also can be nuanced across prescribers.^7^

In response to rising prescription drug prices^8,9^ and DTCA,^1^ the US Department of Health and Human Services attempted regulation^10^ that would require drug manufacturers to include prices in all television advertisements.^11^ More recent bipartisan efforts have attempted to legislate price disclosure requirements in DTCA.^12^ Price disclosure might discourage consumers from seeking treatment with high advertised prices. Alternatively, disclosure may be ineffective if consumers cannot evaluate prescription drug prices or if they view price as a signal of quality. Preliminary research suggests that price disclosure can influence consumer responses,^13^ but the implications of price disclosure on a broad range of behavioral outcomes is unclear.

In this cross-sectional study with a survey, we aim to fill these knowledge gaps by examining the association of DTCA and price disclosure in DTCA with medication- and lifestyle-related intentions, health-related beliefs, and brand perceptions among individuals at risk of heart disease. We focused on those with or at risk of cardiovascular disease, including coronary artery disease, stroke, heart failure, and hypertension.^14^ Cardiovascular disease is highly prevalent in the US and has several pharmacological treatments^15^ and nonpharmacological lifestyle interventions available to patients.^16^ We hypothesized that exposure to DTCA for heart products would increase the likelihood of medication adherence, promote conversations with clinicians, and decrease the likelihood of diet and exercise intentions. We hypothesized that there would be no or small associations with price disclosure.^8,9,10^ As efforts to regulate DTCA continue in Congress, this study will help manufactures, policy makers, and consumers understand the behavioral consequences of DTCA, as well as if price disclosure promotes competitive pressures that could lead to lower prices.

## Methods

### Data and Procedures

Study participants were recruited from Ipsos Public Affairs (hereafter, Ipsos) KnowledgePanel, a national probability-based household panel. The target population consisted of individuals who were at high risk of cardiovascular disease. Recruited participants were English-speaking US residents aged 40 to 64 years who were diagnosed with high cholesterol or currently a cigarette smoker, or overweight or obese (ie, body mass index >25 [calculated as weight in kilograms divided by height in meters squared]). Randomization occurred at the level of the individual into 1 of 3 study arms: exposure to DTCA (treatment 1), exposure to DTCA with prices (treatment 2), and nonpharmaceutical advertising (control group).

Participants in each study arm viewed five 1-minute video advertisements, totaling 5 minutes of advertising exposure. The treatment 1 arm viewed DTCA videos for 4 heart disease medications (Brilinta [AstraZeneca], Entresto [Novartis], Repatha [Amgen], and Xarelto [Bayer]). The advertisements viewed by the treatment 2 arm were identical to the advertisements viewed by the treatment 1 arm, except that treatment 2 arm’s advertising videos were edited to disclose the net prices received by the manufacturer as reported in the SSR Health database.^17^ The control arm viewed nonpharmaceutical advertisements, such as for-consumer electronics and web-based services. The advertisements watched by each study arm were not artificially created by the researchers; instead, they represented advertisements that have been aired on television.

After viewing the advertisements, participants completed a survey questionnaire to measure medication- and lifestyle-related intentions. In addition, participants were asked about their health-related beliefs and brand perceptions. Participants also answered demographic questions and received $5 as compensation for participation. The survey instrument is provided in the eAppendix in the Supplement.

The survey was fielded over a 15-day period from July 21, 2021, to August 4, 2021. Statistical analysis was performed from September 2021 to November 2021. The survey was fielded to 4933 respondents, of which 3026 respondents started and completed the survey, yielding a response rate of 61%. Of the 3026 respondents who completed the survey, 152 respondents did not meet the survey inclusion criteria. The final analytic sample included 2874 respondents across the control arm (n = 952), treatment 1 arm (n = 964), and treatment 2 arm (n = 958).

This study received institutional review board approval from the University of Southern California. All participants provided written informed consent via electronic form. This study follows the Strengthening the Reporting of Observational Studies in Epidemiology (STROBE) reporting guideline^18^ and the American Association for Public Opinion Research reporting guideline for survey studies.^19^

### Measures

To ask about medication-related intentions, we focused on the current pharmacological treatment guidelines that support the use of antihypertensives and statins for prevention of heart disease.^20,21^ Specifically, we relied on prior research to develop survey questions regarding medication-related outcomes that included ordinal measures (ranging from 1 [highly unlikely] to 5 [highly likely]) on the likelihood of switching medication, asking a physician about advertised medication, asking an insurer about advertised medication, searching for medication online, or taking medication as directed.^13,22,23^

To ask about lifestyle-related intentions, we focused on nonpharmacological lifestyle interventions that have been recommended to improve outcomes in individuals at high risk of cardiovascular diseases.^24,25,26^ Specifically, lifestyle-related outcomes included ordinal measures (ranging from 1 [highly unlikely] to 5 [highly likely]) on the likelihood of being more physically active and eating healthier food.

To ask about brand perceptions, we relied on the marketing literature to identify measures that represent consumers’ beliefs about brands as intentional agents.^27^ Consumer beliefs about brands, such as brand competence, innovativeness, and trustworthiness, can guide consumers’ demand intentions (eg, intention to search for medication online), as well as actual behaviors (eg, asking a physician about advertised medication). We included ordinal measures (ranging from 1 [always disagree] to 5 [always agree]) on individual perceptions of pharmaceutical manufacturers as being competent, innovative, and trustworthy.

Finally, to ask about health-related beliefs, we relied on the Determinants of Lifestyle Behavior Questionnaire, a survey instrument that has been shown to be valid for measuring determinants of lifestyle behavioral change in adults at high risk of cardiovascular diseases.^28^ Questions on health-related beliefs were categorized into 2 categories: (1) questions that ask about perceived *importance* of physical activity/dietary behavior and (2) questions that ask about perceived *difficulty* of physical activity/dietary behavior. The perceived importance measure included ordinal measures (ranging from 1 [always disagree] to 5 [always agree]) on beliefs related to physical activity and dietary behavior (eg, “Eating healthier food is pleasant,” “Eating healthier food is important,” “Eating healthier food is easy”). The perceived difficulty measure included ordinal measures (ranging from 1 [always disagree] to 5 [always agree]) on beliefs related to behavior-specific situations (eg, “I am able to eat healthier food on average,” “I find it difficult to eat healthier food on average,” “I find it difficult to eat healthier food when I am busy,” “My family and friends think I should eat healthier food”). We also asked questions on medication-related beliefs, including perceived seriousness of heart disease and perceived effectiveness of heart disease medication.

### Statistical Analysis

Baseline characteristics and outcomes of interest were compared for the treatment and control groups using χ^2^ tests. We estimated the association between DTCA exposure and outcomes of interest using ordered logit regression models.

We conducted 3 sets of regression models. First, we estimated the association of being randomized to a treatment group (ie, either treatment 1 or treatment 2) compared with those who were randomized to the control group. We separately also analyzed those who were randomized to receive treatment 1 (DTCA without price disclosure) compared with the control group and those who were randomized to receive treatment 2 (DTCA with price disclosure) compared with the control group. The key independent variable was an indicator variable for random assignment to a treatment group. All regressions included self-reported demographic characteristics, including participant age, sex, race, household income, and indicators for which of the inclusion criteria the respondent met (ie, high cholesterol, current smoker, overweight or obese).

To assess the magnitude of DTCA associations, we calculated marginal effects (MEs) of treatment holding all other variables at their mean. The MEs can be interpreted as the difference in probability of an outcome between treatment and control arms.

It is possible that the study participants were not representative of individuals at risk for cardiovascular disease because they were much more likely to spend time taking paid surveys. While recent work suggests that Ipsos has lower bias and superior data quality when compared with other surveys,^29^ to gain confidence in the present results, we reestimated all models with the Ipsos-generated survey weights to make the sample comparable with the Current Population Survey.

Given the large number of outcomes, we also estimated Romano-Wolf *P* values by estimating a familywise error rate or the probability of making any type I error.^30^ Stata, version 16.1 (StataCorp) was used for all analyses.

## Results

Overall, observable characteristics were similar across the treatment and control groups (Table 1). The χ^2^ tests demonstrated no statistically significant differences on age, gender, race, household income, education, or census region (eTable 1 in the Supplement).

**Table 1.** Balance in Baseline Covariates Across Treatment and Control Groups^a^

Characteristic	No. (%)			*P* value^b^
	Total (n = 2874)	Control (n = 952)	Treatment (n = 1922)	
Respondent screening, mean (SD)				
High cholesterol	0.52 (0.50)	0.52 (0.50)	0.52 (0.50)	.80
Current smoker	0.16 (0.36)	0.16 (0.36)	0.16 (0.37)	.82
BMI overweight	0.91 (0.28)	0.91 (0.29)	0.91 (0.28)	.76
Age, y				
30-44	443 (15)	134 (14)	309 (16)	.11
45-59	1675 (58)	547 (57)	1128 (59)	
≥60	756 (26)	271 (28)	485 (25)	
Gender				
Female	1325 (46)	459 (48)	866 (45)	.11
Male	1549 (54)	493 (52)	1056 (55)	
Race				
American Indian or Alaska Native	26 (1)	10 (1)	16 (1)	.99
Asian	82 (3)	26 (3)	56 (3)	
Black or African American	290 (10)	99 (10)	191 (10)	
Native Hawaiian or Pacific Islander	3 (0)	1 (0)	2 (0)	
White	2379 (83)	784 (82)	1595 (83)	
≥2 Races	94 (3)	32 (3)	62 (3)	
Household income, $				
<10 000	73 (3)	28 (3)	45 (2)	.12
10 000-24 999	218 (8)	57 (6)	161 (8)	
25 000-49 999	427 (15)	135 (14)	292 (15)	
50 000-74 999	439 (15)	157 (16)	282 (15)	
75 000-99 999	424 (15)	129 (14)	295 (15)	
100 000-149 999	635 (22)	216 (23)	419 (22)	
≥150 000	658 (23)	230 (24)	428 (22)	
Education				
No high school diploma or GED	146 (5)	46 (5)	100 (5)	.59
High school graduate or the equivalent	773 (27)	271 (28)	502 (26)	
Some college or associate’s degree	941 (33)	303 (32)	638 (33)	
Bachelor’s degree or higher	1014 (35)	332 (35)	682 (35)	
US region				
Northeast	538 (19)	179 (19)	359 (19)	.66
Midwest	640 (22)	211 (22)	429 (22)	
South	1063 (37)	364 (38)	699 (36)	
West	633 (22)	198 (21)	435 (23)	

Abbreviations: BMI, body mass index; GED, general education development.

^a^ Respondent-level demographic characteristics at baseline are reported for respondents randomized into the treatment 1 arm (exposure to direct-to-consumer advertising) and the treatment 2 arm (exposure to direct-to-consumer advertising with prices), as well as the control arm (exposure to nonpharmaceutical advertising).

^b^ *P* values reported result from χ^2^ tests.

**Figure 1.**
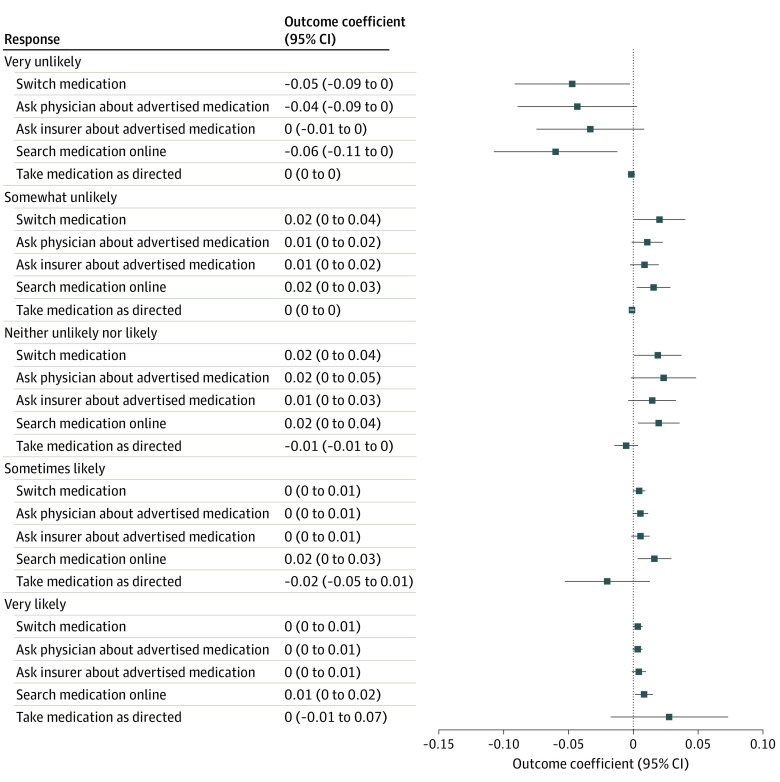
Marginal Effect of Treatment Exposure on Consumer Medication-Related Intentions Presented are marginal effects estimated from ordered logit regressions of each outcome measure on treatment assignment, controlling for respondent age, sex, race, household income, and indicators for which of the inclusion criteria the respondent met (ie, high cholesterol, current smoker, or overweight or obese). The ordered logit regressions estimated the effects of being randomized to either treatment group (ie, either treatment 1 or treatment 2) compared with those who were randomized to the control group. Outcome measures are medication-related outcomes that include ordinal measures (ranging from 1 [highly unlikely] to 5 [highly likely]) on the likelihood of switching medication, asking a physician about advertised medication, asking an insurer about advertised medication, searching for medication online, or taking medication as directed.

Figure 1 illustrates the direct associations of DTCA exposure with medication-related intentions. Respondents in the treatment group reported stronger intentions to search for medications online or to switch medications. For example, respondents in the treatment groups were more likely to report that they were “very likely” to switch medication (ME = 0.004; *P* = .002) and less likely to report that they were “very unlikely” to switch medications (ME = −0.05; *P* = .03). Similarly, respondents in the treatment groups were more likely to report they were “very likely” to search for information about the medication online (ME = 0.02; *P* = .01) and less likely to report that they were “very unlikely” to search for information about the medication online (ME = −0.06; *P* = .01). There was no statistically significant association for the likelihood of taking medication as directed and asking a physician or insurer about the advertised medication.

eFigure 1 in the Supplement displays the indirect associations of DTCA exposure with behavioral intentions (ie, intention to be more physically active and eat healthier food). There were no statistically significant associations of DTCA on these behavioral intentions.

**Figure 2.**
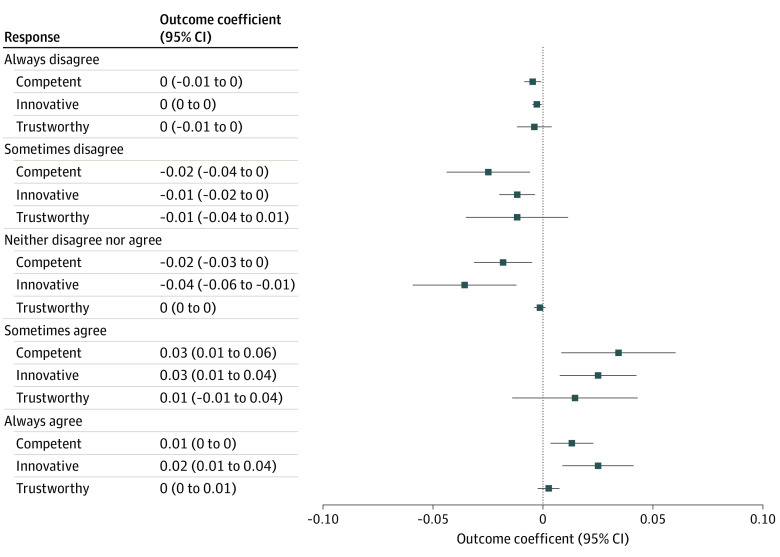
Marginal Effect of Treatment on Consumer Brand Perceptions Presented are marginal effects estimated from ordered logit regressions of each outcome measure on treatment assignment, controlling for respondent age, sex, race, household income, and indicators for which of the inclusion criteria the respondent met (ie, high cholesterol, current smoker, or overweight or obese). The ordered logit regressions estimated the effects of being randomized to either treatment group (ie, either treatment 1 or treatment 2) compared with those who were randomized to the control group). Outcome measures represent consumer beliefs about pharmaceutical manufacturers and include ordinal measures (ranging from 1 [always disagree] to 5 [always agree]) on perceptions of pharmaceutical manufacturers as being competent, innovative, and trustworthy.

Figure 2 illustrates the association between DTCA exposure and perceptions of pharmaceutical manufacturers. Respondents in the treatment groups were more likely to hold beliefs that pharmaceutical firms were competent and innovative. For example, respondents in the treatment groups were likely to “always agree” with the statement that pharmaceutical firms were competent (ME = 0.03; *P* = .01) and innovative (ME = 0.03; *P* = .008). There were no statistically significant differences between the treatment and control groups in perceptions about trustworthiness of pharmaceutical manufacturers. Similarly, there were no differences between the treatment and control groups in perceptions about the competence, innovativeness, and trustworthiness of nonpharmaceutical industries (eFigure 4 in the Supplement).

**Figure 3.**
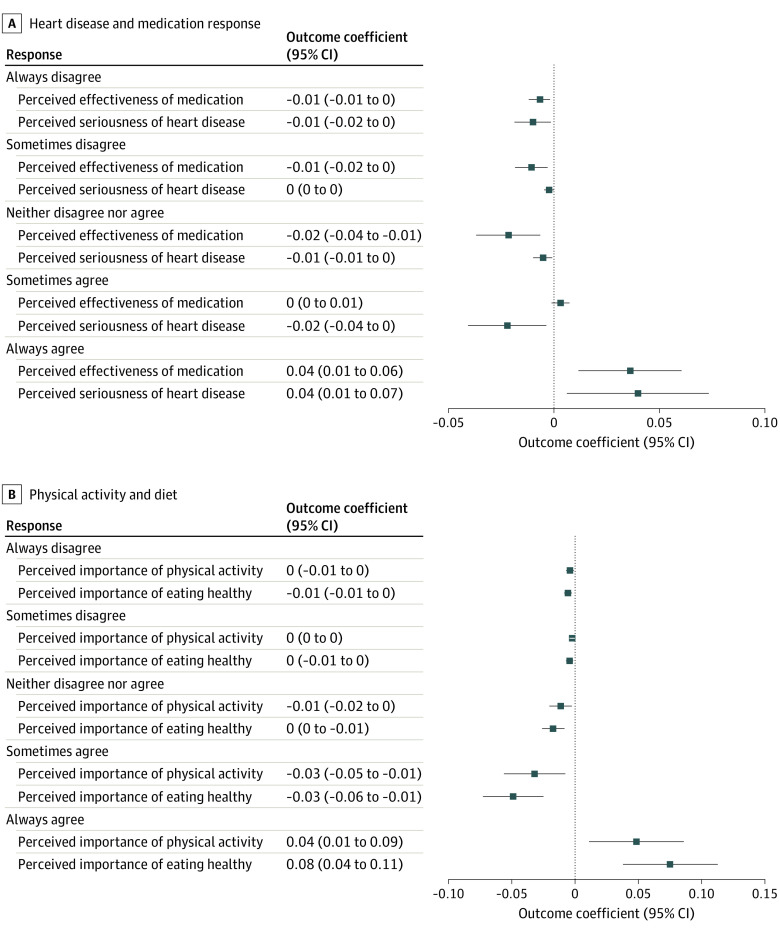
Marginal Effect of Treatment on Consumer Health-Related Beliefs Presented are marginal effects estimated from ordered logit regressions of each outcome measure on treatment assignment, controlling for respondent age, sex, race, household income, and indicators for which of the inclusion criteria the respondent met (ie, high cholesterol, current smoker, or overweight or obese). The ordered logit regressions estimated the effects of being randomized to either treatment group (ie, either treatment 1 or treatment 2) compared with those who were randomized to the control group. Outcome measures include questions on health-related beliefs related to medication, physical activity, and diet. Medication-related questions include ordinal measures (ranging from 1 [always disagree] to 5 [always agree]) on perceived seriousness of heart disease and perceived effectiveness of heart disease medication (A). Measures related to the perceived importance of physical activity and diet include ordinal measures (ranging from 1 [always disagree] to 5 [always agree]) on beliefs related to physical activity and dietary behavior (eg, “Eating healthier food is important,” “Physical activity is important”) (B).

Figure 3 illustrates the associations between DTCA exposure and health-related beliefs across 2 categories: (1) medication and (2) physical activity and diet. Figure 3A shows results for medication-related beliefs, defined as perceived effectiveness of medication and perceived seriousness of heart disease. Respondents exposed to pharmaceutical DTCA were more likely to hold beliefs that medication is an effective treatment for heart disease and that heart disease is a serious condition. In particular, respondents in the treatment groups were statistically significantly more likely to “always agree” with the statement that medication is an effective treatment for heart disease (ME = 0.04; *P* = .003) and the statement that heart disease is serious (ME = 0.04; *P* = .02).

Figure 3B shows the associations between DTCA exposure and physical activity– and diet-related beliefs, defined as the perceived importance of physical activity and healthy eating. Respondents exposed to DTCA were more likely to have favorable perceptions of the importance of physical activity and healthy eating, with no changes to perceived ability to engage in these activities. In particular, respondents exposed to DTCA were more likely to report that they “always agree” with the statement that it is important to be physically active (ME = 0.05, *P* = .01) and eat healthily (ME = 0.08; *P* < .001). We found similar results for perceived pleasantness of physical activity and healthy eating (eFigures 4 and 5 in the Supplement). There were no differences around respondents’ perceived ability to engage in physical activity or healthy eating (eFigure 5 in the Supplement).

There were no statistically significant differences across the 2 treatment arms that did and did not receive price disclosure for outcomes related to medication-related intentions (eFigure 2 in the Supplement), indirect behavioral intentions (eFigure 3 in the Supplement), perceptions of pharmaceutical manufacturers (eFigure 4 in the Supplement), or health-related beliefs (eFigure 5 in the Supplement). eFigure 6 in the Supplement shows similar results when reestimating models with Ipsos-generated survey weights. eTable 6 in the Supplement summarizes Romano-Wolf *P* values for multiple testing. While these adjustments limit the ability to claim statistical significance in the present outcomes, many outcomes have been tested, and this adjustment leans on the conservative side. Finally, eFigure 7 in the Supplement presents regression results without demographic controls, which are consistent with descriptive unadjusted distributions for all outcomes (eTables 2, 3, 4, and 5 in the Supplement).

## Discussion

This cross-sectional study with a survey found that brief exposure to pharmaceutical DTCA has positive, if nuanced, associations around medication-related demand intentions. Direct-to-consumer advertising was associated with increased searching for information but no increased likelihood of physician conversations. This is unsurprising and confirms prior research examining the associations of DTCA in observational settings.^2,3,4,5,6^ These advertisements are commercial in nature, and their goal is to increase the sales of the advertised drug. Most shifts in intentions came from the “very unlikely” category, suggesting that consumers may be more likely to shift away from previously held negative beliefs than positive ones.^31^

We found mixed evidence around diet and exercise. On one hand, we found that exposure to DTCA was not associated with intention to engage in exercise or eat a healthy diet. On the other hand, we found that DTCA was associated with more favorable beliefs about diet and exercise. These results were smaller in magnitude and with mixed statistical significance when compared with the results around medication. Overall, the results suggest that exposure to DTCA for prescription drugs is unlikely to have an adverse effect on diet and exercise. Prior research has found that DTCA generally emphasizes drug promotion over general health education or healthy behaviors.^32^ However, a substantial proportion of DTCA, including advertisements used in this study, highlights healthy lifestyle behaviors. Prior research using observational data has found a similarly mixed picture of the associations of DTCA for prescription drugs with health-related behaviors.^5^ It is also possible that DTCA has a societal-level cumulative effect on these beliefs because those in the treatment and control groups have been exposed to DTCA for several decades. While we found no association of a single exposure to DTCA, this does not mean that DTCA has not affected this population.

We found no statistically significant differences across the treatment arms that did and did not receive price disclosure, in contrast with prior results from Garrett et al^13^ examining the influence of DTCA price disclosure on consumer decision-making.^13^ While the present study focused on the most commonly advertised products for heart disease, regardless of cost, Garrett et al focused on a fictitious diabetes drug with a very high price ($15 500 per month). The difference in results suggests that price disclosure may be more likely to shift consumer thinking when it comes to very high-priced drugs and that the information would be less salient for more moderately priced drugs. These results are in line with the broader literature on price transparency in health care, which suggests that public information does not shift demand in appreciable ways.^33,34,35,36^ The bipartisan Drug-price Transparency for Competition Act is currently pending before Congress.^12^ The present results suggest that consumers are not strongly influenced by price disclosure for moderately priced drugs, and policy makers may wish to consider alternative strategies for promoting competition in this space.

### Limitations

This study has limitations. First, this was an online survey in which study participants viewed advertisements in one sitting. In reality, consumers are exposed to DTCA throughout a variety of media (scrolling through a smartphone, watching television/streaming services, and banner/video advertisements on the web). Despite this artificial setting, the present results have internal validity (owing to the randomized nature of the survey) and a case for generalizability because we recruited a large sample (n = 2874) that was nationally representative of the US population with or at risk for heart disease. Second, the study was unable to address actual demand for prescription drugs and instead had to focus on beliefs and intentions. This may be particularly worrisome in the health care context, where the link between behavioral intentions and eventual behavior change can be weak.^37^ Third, the study focused on only the short-term behavioral intentions even though advertising is known to have both an instantaneous (flow) and long-term accumulation and depreciation (stock) effect. Future research should focus on the long-term effects of advertising in a real-world, randomized setting. Fourth, the study lacks a true control group because pharmaceutical DTCA has been ubiquitous in the US for several decades. Fifth, the analysis only examined DTCA relative to advertisements for nonhealth consumer products. The results may be explained by priming effects, and we might have seen similar effects if the treatment group was exposed to advertisements for healthy foods. Lastly, we focused only on 5 advertisements for a single product category (heart disease), limiting generalizability to other conditions.

## Conclusion

Results of this cross-sectional study with a survey show that brief exposure to pharmaceutical DTCA has a large and positive association on medication-related demand intentions with no offsetting negative spillovers on lifestyle-related intentions. These results suggest that sustained expansion of DTCA in the US can have important demand effects on consumer behavior. This study indicates that the threat that DTCA may generate welfare-reducing lifestyle changes is not supported by the data. Moreover, the present results suggest DTCA might influence consumer views on policies related to pharmaceutical manufacturers. Lack of associations with price disclosure in DTCA suggests that policy makers should consider alternative strategies to promote value-based decision-making for prescription drugs.

## Notes

Supplement. **eAppendix.** Survey Instrument **eTable 1.** Balance in Baseline Covariates Across Treatment Groups **eFigure 1.** Marginal Effect of Treatment Exposure on Indirect Behavioral Intentions **eFigure 2.** Marginal Effect of Treatment Exposure on Medication-Related Intentions, By Treatment Arm **eFigure 3.** Marginal Effect of Treatment Exposure on Indirect Behavioral Intentions, By Treatment Arm **eFigure 4.** Marginal Effect of Treatment on Brand Perceptions **eFigure 5.** Marginal Effect of Treatment on Health-Related Beliefs, By Treatment Arm **eFigure 6.** Marginal Effect of Treatment on Medication-Related Intentions, Indirect Behavioral Intentions, Brand Perceptions, and Health-Related Beliefs (weighted regressions) **eFigure 7.** Marginal Effect of Treatment on Medication-Related Intentions, Indirect Behavioral Intentions, Brand Perceptions, and Health-Related Beliefs (without demographic controls) **eTable 2.** Descriptive Statistics for Medication-Related Intentions, By Treatment Arm **eTable 3.** Descriptive Statistics for Indirect Behavioral Intentions, By Treatment Arm **eTable 4.** Descriptive Statistics for Brand Perceptions, By Treatment Arm **eTable 5.** Descriptive Statistics for Health-Related Beliefs, By Treatment Arm **eTable 6.** Unadjusted and Romano-Wolf *P* values After Multiplicity Adjustment
