# Supplementary material for: Association of Direct-to-Consumer Advertising of Prescription Drugs With Consumer Health-Related Intentions and Beliefs Among Individuals at Risk of Cardiovascular Disease
Source: JAMA Health Forum. 2022 Aug 12;3(8):e222570. doi: 10.1001/jamahealthforum.2022.2570 (PMC9375162; doi:10.1001/jamahealthforum.2022.2570)
Supplement: Supplement. — eAppendix. Survey Instrument eTable 1. Balance in Baseline Covariates Across Treatment Groups eFigure 1. Marginal Effect of Treatment Exposure on Indirect Behavioral Intentions eFigure 2. Marginal Effect of Treatment Exposure on Medication-Related Intentions, By Treatment Arm eFigure 3. Marginal Effect of Treatment Exposure on Indirect Behavioral Intentions, By Treatment Arm eFigure 4. Marginal Effect of Treatment on Brand Perceptions eFigure 5. Marginal Effect of Treatment on Health-Related Beliefs, By Treatment Arm eFigure 6. Marginal Effect of Treatment on Medication-Related Intentions, Indirect Behavioral Intentions, Brand Perceptions, and Health-Related Beliefs (weighted regressions) eFigure 7. Marginal Effect of Treatment on Medication-Related Intentions, Indirect Behavioral Intentions, Brand Perceptions, and Health-Related Beliefs (without demographic controls) eTable 2. Descriptive Statistics for Medication-Related Intentions, By Treatment Arm eTable 3. Descriptive Statistics for Indirect Behavioral Intentions, By Treatment Arm eTable 4. Descriptive Statistics for Brand Perceptions, By Treatment Arm eTable 5. Descriptive Statistics for Health-Related Beliefs, By Treatment Arm eTable 6. Unadjusted and Romano-Wolf P values After Multiplicity Adjustment [file jamahealthforum-e222570-s001.pdf]

# Supplemental Online Content

Eisenberg MD, Singh Y, Sood N. Association of direct-to-consumer advertising of prescription drugs with consumer health-related intentions and beliefs among individuals at risk of cardiovascular disease. *JAMA Health Forum*. 2022;3(8):e222570. doi:10.1001/jamahealthforum.2022.2570

**eAppendix.** Survey Instrument

**eTable 1.** Balance in Baseline Covariates Across Treatment Groups

**eFigure 1.** Marginal Effect of Treatment Exposure on Indirect Behavioral Intentions

**eFigure 2.** Marginal Effect of Treatment Exposure on Medication-Related Intentions, By Treatment Arm

**eFigure 3.** Marginal Effect of Treatment Exposure on Indirect Behavioral Intentions, By Treatment Arm

**eFigure 4.** Marginal Effect of Treatment on Brand Perceptions

**eFigure 5.** Marginal Effect of Treatment on Health-Related Beliefs, By Treatment Arm

**eFigure 6.** Marginal Effect of Treatment on Medication-Related Intentions, Indirect Behavioral Intentions, Brand Perceptions, and Health-Related Beliefs (weighted regressions)

**eFigure 7.** Marginal Effect of Treatment on Medication-Related Intentions, Indirect Behavioral Intentions, Brand Perceptions, and Health-Related Beliefs (without demographic controls)

**eTable 2.** Descriptive Statistics for Medication-Related Intentions, By Treatment Arm

**eTable 3.** Descriptive Statistics for Indirect Behavioral Intentions, By Treatment Arm

**eTable 4.** Descriptive Statistics for Brand Perceptions, By Treatment Arm

**eTable 5.** Descriptive Statistics for Health-Related Beliefs, By Treatment Arm

**eTable 6.** Unadjusted and Romano-Wolf *P* values After Multiplicity Adjustment

This supplemental material has been provided by the authors to give readers additional information about their work.

**eAppendix. Survey Instrument**

## Introduction

Base: all respondents

**DISP1[DISP]**

Thank you very much for participating in KnowledgePanel® surveys. This particular survey focuses on health communications and will take about 10 minutes to complete.

This survey is being conducted by University of Southern California. The study will help researchers understand how advertising affects attitudes and behaviors.

As with all KnowledgePanel® surveys, responding to this survey, or to any individual question on the survey, is completely voluntary. Your responses remain anonymous and will be used for research analyses only.

There are no risks from participating in this study. The possible benefits to you will include 5,000 bonus points (worth $5) if you qualify and complete today's survey. Based on your answers, you may qualify for two brief follow up surveys coming in the next few weeks on the same topic. You will earn an additional 10,000 bonus points if you are selected and complete both of the follow-up surveys.

If you have questions about your rights as a participant in this survey, or are dissatisfied at any time with any aspect of the survey, you may contact the KnowledgePanel Panel Member Support at 800-782-6899.

If you consent to participate in this study, please click the NEXT button below to complete the survey.

Base: all respondents
Prompt once

**CHLEV** [S]

Have you EVER been told by a doctor or other health professional that you had high cholesterol?

1 Yes

2 No

Base: all respondents
Prompt once

**SMKEV** [S]

Have you smoked at least 100 cigarettes in your ENTIRE LIFE?

© 2022 Eisenberg MD et al. *JAMA Health Forum.*

1 Yes

2 No

Base: IF smkev=1
Prompt once

**SMKNOW** [S]

Do you NOW smoke cigarettes every day, some days or not at all?

1 Every day

2 Some days

3 Not at all

Base: all respondents
Prompt once

**Height [Q]**

How tall are you without shoes?

*Please type in the number of feet and inches separately. For example, if you are 6' 0" tall, type 6 in the feet box and 0 in the inches box.*

Base: all respondents
Prompt once

**Weight [Q]**

How much do you weigh without shoes?

Base: all respondents

**S0 [DISP]**

In this survey, we will be showing videos and asking for your responses. Please make sure your volume is at an audible level. Please be patient while the videos load and press "play" once you see the play icon appear.

**[SAMPLE VIDEO]**

Base: all respondents

**S1 [M]**

Were you able to view or hear this video?

© 2022 Eisenberg MD et al. *JAMA Health Forum.*

1. Yes **[S]**
2. No, I could not view the video
3. No, I could not hear the video

*PROGRAMMER: PROMPT ONCE IF REFUSED. TERMINATE IF S1=2,3, REFUSED.*

Base: all respondents

*PROGRAMMER: RANDOMLY ASSIGN RESPONDENTS INTO 3 TREATMENT ARMS*

*DOV_ARM [S]:*

1. *Control*
2. *Treatment 1 (T1)*
3. *Treatment 2 (T2)*

*RANDOMIZE THE ORDER OF 5 VIDEOS WITHIN ARMS*

*DOV_VIDEO [S]*

1. *Control - eharmony Video Date First Date Promotional Video*
2. *Control - GEICO Homeowners Insurance TV Commercial, Overflowing Office*
3. *Control - Princess Dress Tide*
4. *Control - T-Mobile Commercial 2017 - (USA)*
5. *Control - xrIphone New AD of Apple Face ID Security on Nap*
6. *T1 - Brilinta (Hide Price)-converted*
7. *T1 - Enresto 2 (Hide Price)-converted*
8. *T1 - Entresto (Hide Price)-converted*
9. *T1 - Repaetha (Hide Price)-converted*
10. *T1 - Xarelto (Hide Price)-converted*
11. *T2 - Brilinta (New Price)-converted*
12. *T2 - Enresto 2 (New Price)-converted*
13. *T2 - Entresto (New Price)-converted*
14. *T2 - Repaetha (New Price)-converted*
15. *T2 - Xarelto (New Price)-converted*

Base: DOV_ARM=2,3
Prompt once if refused

**Q1a [S]**

Please watch the following video to completion, then answer the questions that follow.

**[SHOW VIDEO IN THE 1ST ORDER]**

The advertisement you just watched was for a prescription drug that can treat which of the following conditions?

1. Heart Disease
2. Lung Cancer

© 2022 Eisenberg MD et al. *JAMA Health Forum.*

3. Arthritis
4. Type 2 diabetes
5. COVID-19

Base: DOV_ARM=1
Prompt once if refused

**Q1b [S]**

Please watch the following video to completion, then answer the questions that follow.

**[SHOW VIDEO IN THE 1ST ORDER]**

The advertisement you just watched was for which of the following products?

1. Cars
2. Home Insurance
3. Cleaning Detergent
4. Phones
5. Mobile Network
6. Dating Service

Base: DOV_ARM=2,3
Prompt once if refused

**Q2a [S]**

Please watch the following video to completion, then answer the questions that follow.

**[SHOW VIDEO IN THE 2ND ORDER]**

The advertisement you just watched was for a prescription drug that can treat which of the following conditions?

1. Heart Disease
2. Lung Cancer
3. Arthritis
4. Type 2 diabetes
5. COVID-19

Base: DOV_ARM=1
Prompt once if refused

**Q2b [S]**

Please watch the following video to completion, then answer the questions that follow.

**[SHOW VIDEO IN THE 2ND ORDER]**

The advertisement you just watched was for which of the following products?

© 2022 Eisenberg MD et al. *JAMA Health Forum.*

1. Cars
2. Home Insurance
3. Cleaning Detergent
4. Phones
5. Mobile Network
6. Dating Service

Base: DOV_ARM=2,3
Prompt once if refused

**Q3a [S]**

Please watch the following video to completion, then answer the questions that follow.

**[SHOW VIDEO IN THE 3RD ORDER]**

The advertisement you just watched was for a prescription drug that can treat which of the following conditions?

1. Heart Disease
2. Lung Cancer
3. Arthritis
4. Type 2 diabetes
5. COVID-19

Base: DOV_ARM=1
Prompt once if refused

**Q3b [S]**

Please watch the following video to completion, then answer the questions that follow.

**[SHOW VIDEO IN THE 3RD ORDER]**

The advertisement you just watched was for which of the following products?

1. Cars
2. Home Insurance
3. Cleaning Detergent
4. Phones
5. Mobile Network
6. Dating Service

Base: DOV_ARM=2,3
Prompt once if refused

**Q4a [S]**

Please watch the following video to completion, then answer the questions that follow.

**[SHOW VIDEO IN THE 4TH ORDER]**

© 2022 Eisenberg MD et al. *JAMA Health Forum.*

The advertisement you just watched was for a prescription drug that can treat which of the following conditions?

1. Heart Disease
2. Lung Cancer
3. Arthritis
4. Type 2 diabetes
5. COVID-19

Base: DOV_ARM=1
Prompt once if refused

**Q4b [S]**

Please watch the following video to completion, then answer the questions that follow.

**[SHOW VIDEO IN THE 4TH ORDER]**

The advertisement you just watched was for which of the following products?

1. Cars
2. Home Insurance
3. Cleaning Detergent
4. Phones
5. Mobile Network
6. Dating Service

Base: DOV_ARM=2,3
Prompt once if refused

**Q5a [S]**

Please watch the following video to completion, then answer the questions that follow.

**[SHOW VIDEO IN THE 5TH ORDER]**

The advertisement you just watched was for a prescription drug that can treat which of the following conditions?

1. Heart Disease
2. Lung Cancer
3. Arthritis
4. Type 2 diabetes
5. COVID-19

Base: DOV_ARM=1
Prompt once if refused

**Q5b [S]**

© 2022 Eisenberg MD et al. *JAMA Health Forum.*

Please watch the following video to completion, then answer the questions that follow.

**[SHOW VIDEO IN THE 5TH ORDER]**

The advertisement you just watched was for which of the following products?

1. Cars
2. Home Insurance
3. Cleaning Detergent
4. Phones
5. Mobile Network
6. Dating Service

Base: all respondents

*PROGRAMMER: RANDOMIZE THE ORDER OF SECTION I TO V. SHOW SECTIONS PER RANDOM ORDER. SHOW SECTION HEADER ON TOP LEFT CORNER ABOVE QUESTION. E.g.* Medication; Brand; Physical Activity; Diet; Media Consumption;

## SECTION I - MEDICATION

Base: all respondents

**Q6 [M]**

Do you suffer from any of the following chronic conditions?

1. Hypertension
2. High cholesterol
3. Heart disease
4. None of the above **[S]**

Base: Q6=1,2,3

**Q7 [S]**

Are you currently taking any medications for these conditions?

1. Repatha
2. Brilinta
3. Entresto
4. Xarelto
5. Other
6. I am not taking any medication for these chronic conditions

Base: Q7=1-5

**Q8 [S]**

Did you take your medication as directed in the past two weeks?

© 2022 Eisenberg MD et al. *JAMA Health Forum.*

1. Never take as directed
2. Rarely take as directed
3. Sometimes take as directed
4. Frequently take as directed
5. Always take as directed

Base: Q7=1-5

**Q9 [S]**

Do you plan to take your medication as directed over the next two weeks?

1. Never take as directed
2. Rarely take as directed
3. Sometimes take as directed
4. Frequently take as directed
5. Always take as directed

Base: Q7=1-5

**Q10 [S]**

Please indicate the likelihood that you will switch medications in the next two weeks.

1. Very unlikely
2. Somewhat unlikely
3. Neither unlikely nor likely
4. Somewhat likely
5. Very likely

Base: Q6=1,2,3

**Q11 [ACCORDION, S]**

Please indicate the likelihood that you will ask your doctor about medication to treat your chronic heart chronic conditions in the next two weeks.

STATEMENT:

a. Repatha
b. Brilinta
c. Entresto
d. Xarelto

SCALE:

1. Very unlikely
2. Somewhat unlikely

© 2022 Eisenberg MD et al. *JAMA Health Forum.*

3. Neither unlikely nor likely
4. Somewhat likely
5. Very likely

Base: Q6=1,2,3

**Q12 [S]**

Please rate the likelihood that you will contact your insurance provider to ask about coverage for heart disease medication in the next two weeks.

1. Very unlikely
2. Somewhat unlikely
3. Neither unlikely nor likely
4. Somewhat likely
5. Very likely

Base: Q6=1,2,3

**Q13 [S]**

Please rate the likelihood that you will research heart disease medication online in the next two weeks.

1. Very unlikely
2. Somewhat unlikely
3. Neither unlikely nor likely
4. Somewhat likely
5. Very likely

Base: ALL RESPONDENTS

**Q14 [S]**

How effective do you think medication is at treating heart disease?

1. Very ineffective
2. Somewhat ineffective
3. Neither ineffective nor effective
4. Somewhat effective
5. Very effective

Base: ALL RESPONDENTS

**Q15 [S]**

How serious do you think heart disease is?

1. Very unserious
2. Somewhat unserious
3. Neither unserious nor serious
4. Somewhat serious

© 2022 Eisenberg MD et al. *JAMA Health Forum.*

5. Very serious

## SECTION II - BRAND

Base: all respondents

**Q16 [S]**

Please indicate the extent to which you agree with the following statement: Pharmaceutical manufacturers are competent.

1. Always disagree
2. Sometimes disagree
3. Neither disagree nor agree
4. Sometimes agree
5. Always agree

Base: all respondents

**Q17 [S]**

Please indicate the extent to which you agree with the following statement: Pharmaceutical manufacturers are innovative.

1. Always disagree
2. Sometimes disagree
3. Neither disagree nor agree
4. Sometimes agree
5. Always agree

Base: all respondents

**Q18 [ACCORDION, S]**

Please indicate how much you trust businesses in each of the following sectors to do what is right.

STATEMENT:

a. Consumer electronics
b. Online retailers
c. Hospitals/Clinics
d. Insurance
e. Pharmaceuticals

SCALE:

© 2022 Eisenberg MD et al. *JAMA Health Forum.*

1. Always distrust
2. Sometimes distrust
3. Neither distrust nor trust
4. Sometimes trust
5. Always trust

## SECTION III - PHYSICAL ACTIVITY

Base: all respondents

**Q19 [ACCORDION, S]**

In my opinion, being more physically active is… (more physical activity means at least 20 minutes of vigorous exercise at least 3 times per week)

STATEMENT:

a. Pleasant
b. Important
c. Easy

SCALE:

1. Always disagree
2. Sometimes disagree
3. Neither disagree nor agree
4. Sometimes agree
5. Always agree
6.

Base: all respondents

**Q20 [ACCORDION, S]**

Please indicate the extent to which you agree with the following statements.

STATEMENT:

a. I am able to be more physically active on average.
b. I find it difficult to be more physically active on average.
c. I find it difficult to be more physically active when I am busy.
d. I intend to be more physically active in the next two weeks.
e. My family and friends think I should be more physically active.

SCALE:

1. Always disagree
2. Sometimes disagree
3. Neither disagree nor agree

© 2022 Eisenberg MD et al. *JAMA Health Forum.*

4. Sometimes agree
5. Always agree

## SECTION IV - DIET

Base: all respondents

**Q21 [ACCORDION, S]**

In my opinion, eating healthier food is… (eating healthier food means: a diet with less (saturated) fats, more vegetables and fruit, and less calories)

STATEMENT:

a. Pleasant
b. Important
c. Easy

SCALE:

1. Always disagree
2. Sometimes disagree
3. Neither disagree nor agree
4. Sometimes agree
5. Always agree

Base: all respondents

**Q22 [ACCORDION, S]**

Please indicate the extent to which you agree with the following statements.

STATEMENT:

a. I am able to eat healthier food on average.
b. I find it difficult to eat healthier food on average.
c. I find it difficult to eat healthier food when I am busy.
d. I intend to eat healthier food in the next two weeks.
e. My family and friends think I should eat healthier food.

SCALE:

1. Always disagree
2. Sometimes disagree
3. Neither disagree nor agree
4. Sometimes agree
5. Always agree

© 2022 Eisenberg MD et al. *JAMA Health Forum.*

## SECTION V - MEDIA CONSUMPTION

Base: all respondents

**Q23 [S]**

Before today, in the past week, do you recall seeing any pharmaceutical video ads for heart disease?

1. Yes
2. No

Base: IF Q23=1

**Q24 [S]**

On average, how many pharmaceutical video ads for heart disease do you recall seeing in the past week? (including on television, streaming services, etc.)

1. 1-2 ads a week
2. 3-5 ads a week
3. 5- 7 ads a week
4. 7-10 ads a week
5. More than 10 ads a week

## SECTION VI - GENERAL

Base: all respondents

**Q27 [S]**

Do you currently have health insurance?

1. Yes
2. No

Base: if Q27=1

**Q28 [S]**

Is your insurance plan a high-deductible plan?

1. Yes
2. No
3. I am not sure.
4. I do not have health insurance.

Base: all respondents

© 2022 Eisenberg MD et al. *JAMA Health Forum.*

**Q29 [S]**

Does your insurance include prescription medication coverage?

1. Yes
2. No
3. I am not sure.

Base: all respondents

**Q30 [S]**

Please select the option that most accurately describes your tobacco use:

1. Current every day smoker
2. Current some day smoker
3. Former smoker
4. Never smoker

Base: all respondents

**Q31 [S]**

Have you had a usual source of care in the past year? (Usual source of care means the medical professional, doctor's office, clinic, or health center where you would usually go if sick or in need of medical advice)

1. Yes
2. No

Base: all respondents

**DEBRIEF [DISP]**

Thank you for participating in this study. The goal of this study was to study the effects of prescription drug advertising on consumer attitudes and behaviors. This study had two main goals (1) examine the effects of prescription drug advertising on attitudes and behaviors and (2) examine the effects of price disclosure in prescription drug advertising on attitudes and behaviors. The results of this study will be published in peer-reviewed journals. Your participation and responses will remain anonymous.

© 2022 Eisenberg MD et al. *JAMA Health Forum.*

## eTable 1. Balance in Baseline Covariates Across Treatment Groups

| | Total | Control | Treatment Arm Without Price Exposure | Treatment Arm With Price Exposure | p-value |
|---|---|---|---|---|---|
| | N=2,874 | N=952 | N=964 | N=958 | |
| Screening - High Cholesterol | 0.52 (0.50) | 0.52 (0.50) | 0.54 (0.50) | 0.51 (0.50) | 0.64 |
| Screening - Current Smoker | 0.16 (0.36) | 0.16 (0.36) | 0.15 (0.36) | 0.16 (0.37) | 0.80 |
| Screening - BMI Overweight | 0.91 (0.28) | 0.91 (0.29) | 0.92 (0.27) | 0.91 (0.29) | 0.71 |
| Age | | | | | 0.33 |
| 30-44 | 15% | 14% | 16% | 16% | |
| 45-59 | 58% | 57% | 58% | 59% | |
| 60+ | 26% | 28% | 26% | 25% | |
| Gender | | | | | 0.13 |
| Male | 54% | 52% | 54% | 56% | |
| Female | 46% | 48% | 46% | 44% | |
| Race | | | | | 0.97 |
| White | 83% | 82% | 82% | 84% | |
| Black or African American | 10% | 10% | 10% | 10% | |
| American Indian or Alaska Native | 1% | 1% | 1% | 1% | |
| Asian | 3% | 3% | 3% | 3% | |
| Native Hawaiian/Pacific Islander | 0% | 0% | 0% | 0% | |
| 2+ races | 3% | 3% | 3% | 3% | |
| Household Income | | | | | 0.44 |
| Less than $10,000 | 3% | 3% | 2% | 2% | |
| $10,000 to $24,999 | 8% | 6% | 9% | 8% | |
| $25,000 to $49,999 | 15% | 14% | 15% | 16% | |
| $50,000 to $74,999 | 15% | 16% | 15% | 14% | |
| $75,000 to $99,999 | 15% | 14% | 16% | 15% | |
| $100,000 to $149,999 | 22% | 23% | 22% | 21% | |
| $150,000 or more | 23% | 24% | 21% | 23% | |
| Education | | | | | 0.83 |
| No high school diploma or GED | 5% | 5% | 5% | 5% | |

© 2022 Eisenberg MD et al. *JAMA Health Forum.*

| | | | | | |
|---|---|---|---|---|---|
| High school graduate (high school diploma or the equivalent GED) | 27% | 28% | 25% | 27% | |
| Some college or Associate's degree | 33% | 32% | 34% | 33% | |
| Bachelor's degree or higher | 35% | 35% | 36% | 35% | |
| Region | | | | | 0.93 |
| Northeast | 19% | 19% | 19% | 18% | |
| Midwest | 22% | 22% | 22% | 23% | |
| South | 37% | 38% | 36% | 37% | |
| West | 22% | 21% | 23% | 22% | |

Notes: eTable 1 reports respondent-level demographic characteristics at baseline for respondents randomized into Treatment 1 (exposure to DTCA), Treatment 2 (exposure to DTCA with prices), and Control arm (exposure to non-pharmaceutical advertising). P-value reports results from Chi-square tests.

**eFigure 1. Marginal Effect of Treatment Exposure on Indirect Behavioral Intentions**

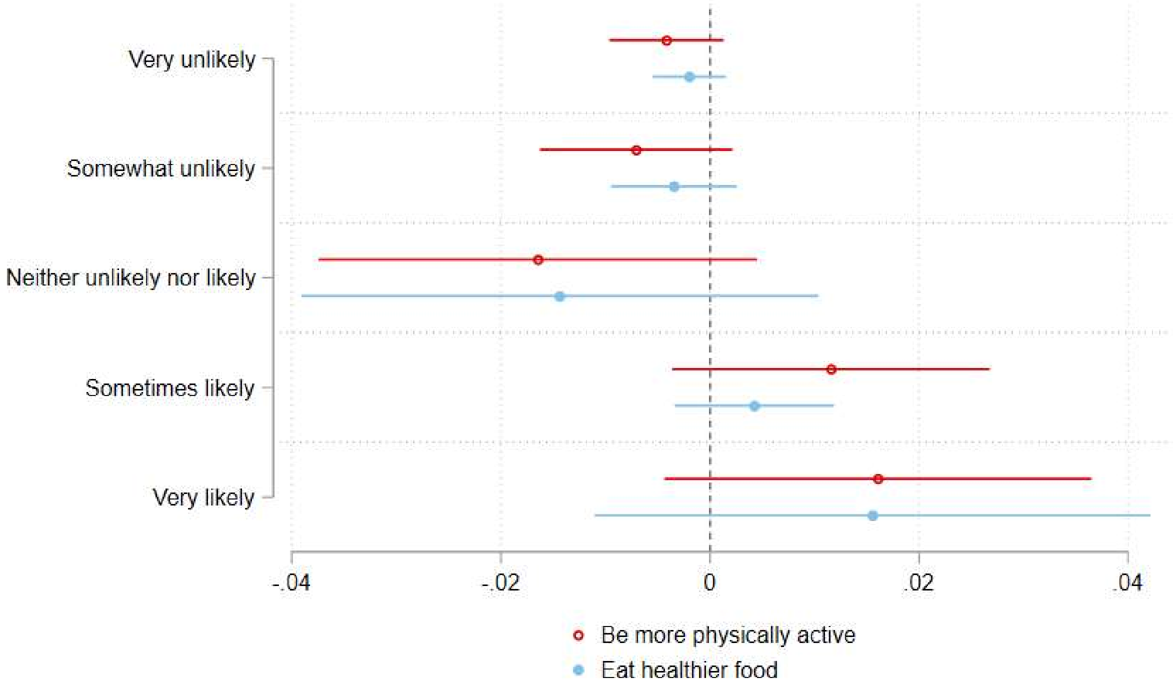


Notes: eFigure 1 presents marginal effects estimated from ordered logit regressions of each outcome measure on treatment assignment, controlling for respondent age, sex, race, household income, and indicators for which of the inclusion criteria the respondent met (i.e., high cholesterol, current smoker, or overweight or obese). The ordered logit regressions estimated the effects of being randomized to either treatment group (i.e., either Treatment 1 or Treatment 2) compared to those who were randomized to the control group). Outcome measures represent non-pharmacological interventions recommended to improve outcomes in individuals at high risk of cardiovascular diseases, and include ordinal measures (ranging from 1 [highly unlikely] to 5 [highly likely]) on the likelihood of being more physically active and eating healthier food.

© 2022 Eisenberg MD et al. *JAMA Health Forum.*

**eFigure 2. Marginal Effect of Treatment Exposure on Medication-Related Intentions, By Treatment Arm**

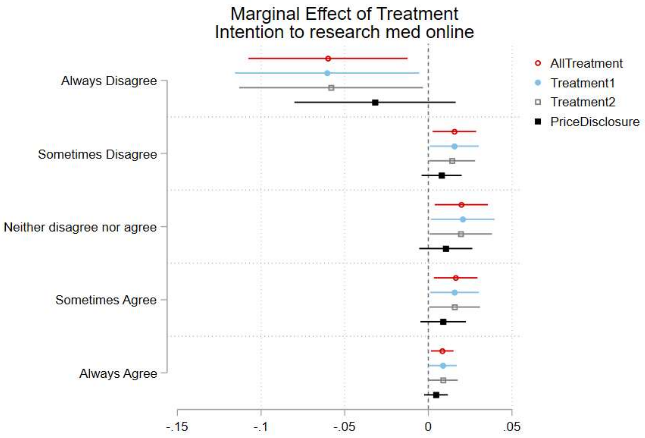


a)

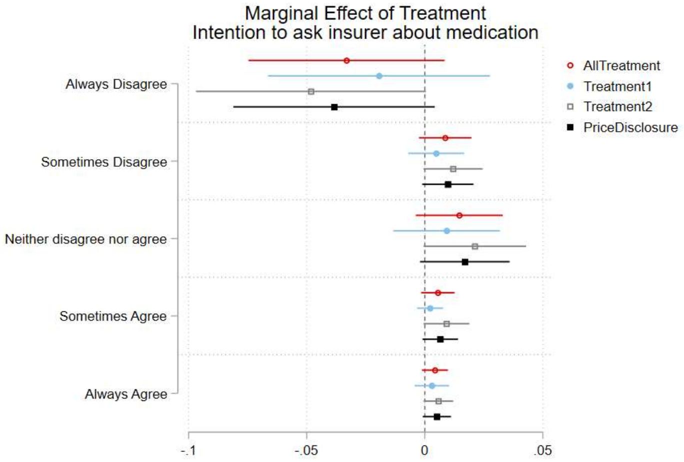


b)

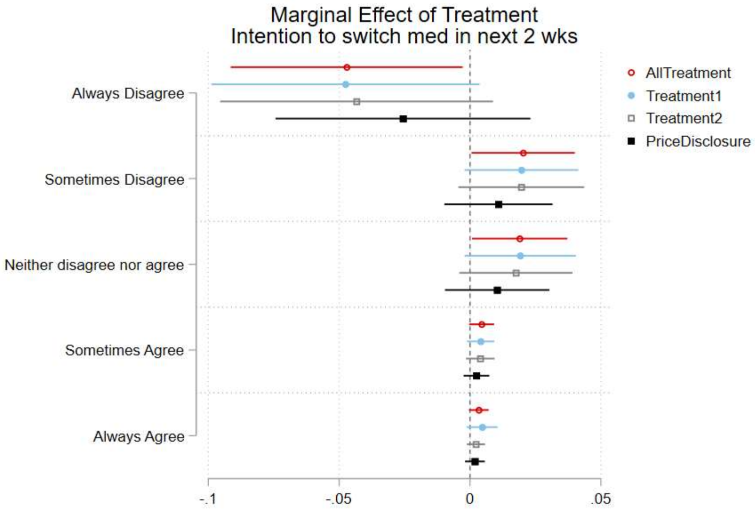


c)

© 2022 Eisenberg MD et al. *JAMA Health Forum.*

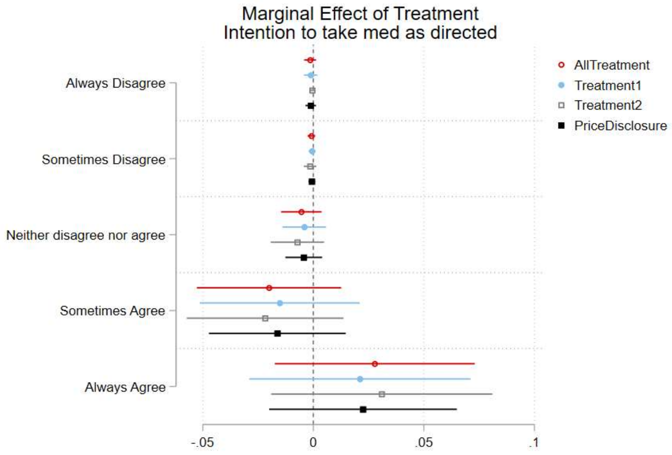


d)

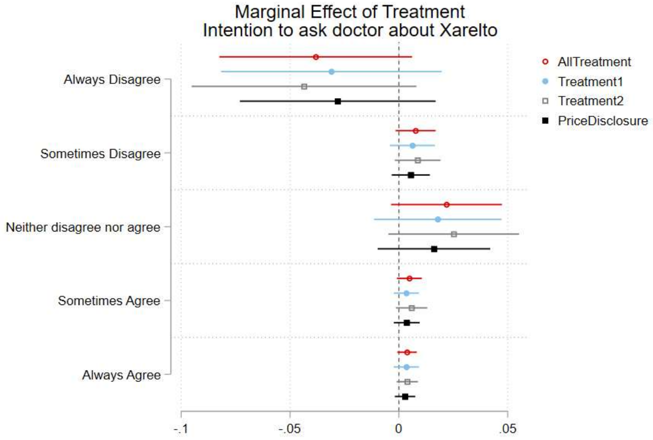


e)

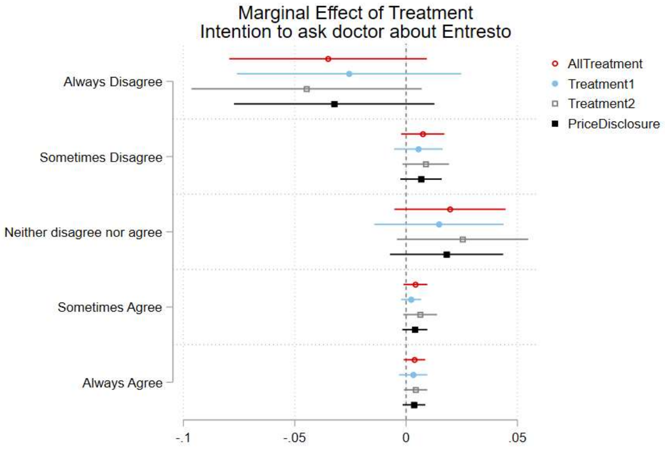


f)

© 2022 Eisenberg MD et al. *JAMA Health Forum.*

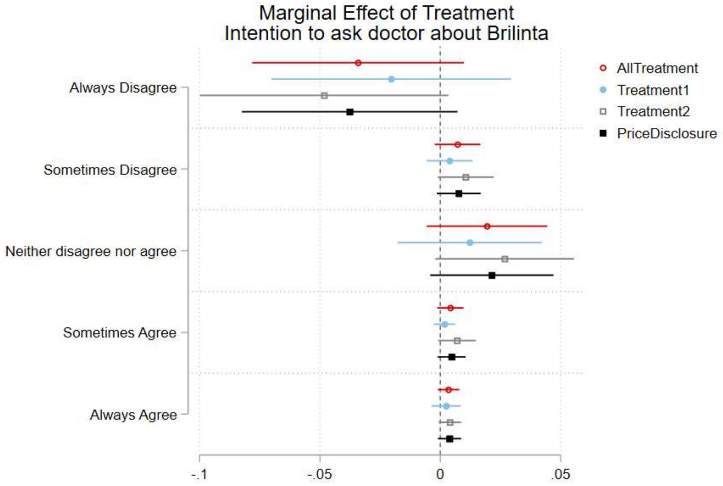


g)

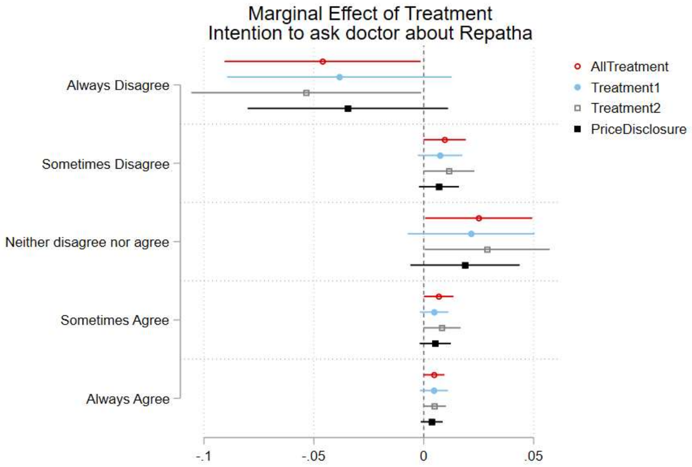


h)

Notes: eFigure 2 presents marginal effects estimated from ordered logit regressions of each outcome measure on treatment assignment, controlling for respondent age, sex, race, household income, and indicators for which of the inclusion criteria the respondent met (i.e., high cholesterol, current smoker, or overweight or obese). The coefficients labeled "All Treatment" represent results from ordered logit regressions that estimated the effects of being randomized to either treatment group (i.e., either Treatment 1 or Treatment 2) compared to those who were randomized to the control group). The coefficients labeled "Treatment 1" represent results from ordered logit regressions that estimated the effects of being randomized to receive Treatment 1 (DTCA without price disclosure) compared to those who were randomized to the control group. The coefficients labeled "Treatment 2" represent results from ordered logit regressions that estimated the effects of being randomized to receive Treatment 2 (DTCA with price disclosure) compared to those who were randomized to the control group. The coefficients labeled "Price Disclosure" compare the coefficients obtained from "Treatment 1" and "Treatment 2" regressions to present the differential effect of price disclosure on outcomes of interest. Outcome measures are medication-related outcomes that include ordinal measures (ranging from 1 [highly unlikely] to 5 [highly likely]) on the likelihood of switching medication, asking doctor about advertised medication, asking insurer about advertised medication, searching for medication online, or taking medication as directed.

© 2022 Eisenberg MD et al. *JAMA Health Forum.*

## eFigure 3. Marginal Effect of Treatment Exposure on Indirect Behavioral Intentions, By Treatment Arm

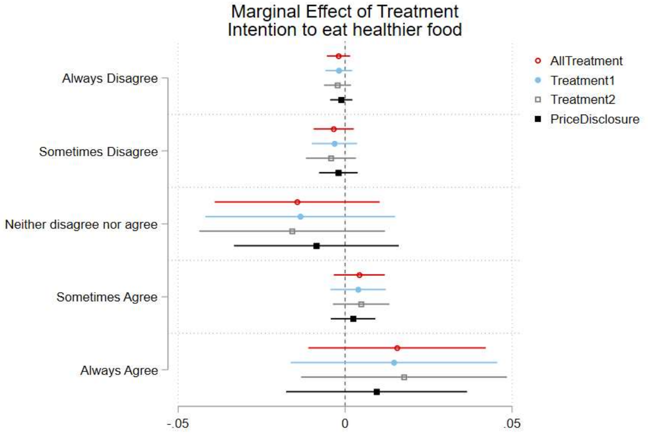


a)

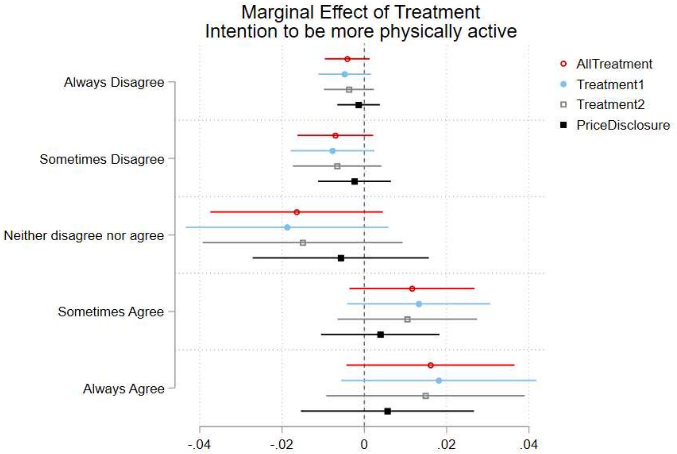


b)

Notes: eFigure 3 presents marginal effects estimated from ordered logit regressions of each outcome measure on treatment assignment, controlling for respondent age, sex, race, household income, and indicators for which of the inclusion criteria the respondent met (i.e., high cholesterol, current smoker, or overweight or obese). The coefficients labeled “All Treatment” represent results from ordered logit regressions that estimated the effects of being randomized to either treatment group (i.e., either Treatment 1 or Treatment 2) compared to those who were randomized to the control group). The coefficients labeled “Treatment 1” represent results from ordered logit regressions that estimated the effects of being randomized to receive Treatment 1 (DTCA without price disclosure) compared to those who were randomized to the control group. The coefficients labeled “Treatment 2” represent results from ordered logit regressions that estimated the effects of being randomized to receive Treatment 2 (DTCA with price disclosure) compared to those who were randomized to the control group. The coefficients labeled “Price Disclosure” compare the coefficients obtained from “Treatment 1” and “Treatment 2” regressions to present the differential effect of price disclosure on outcomes of interest. Outcome measures represent non-pharmacological interventions recommended to improve outcomes in individuals at high risk of cardiovascular diseases and include ordinal measures (ranging from 1 [highly unlikely] to 5 [highly likely]) on the likelihood of being more physically active and eating healthier food.

© 2022 Eisenberg MD et al. *JAMA Health Forum.*

## eFigure 4. Marginal Effect of Treatment on Brand Perceptions

### 4a: Relative to Placebos

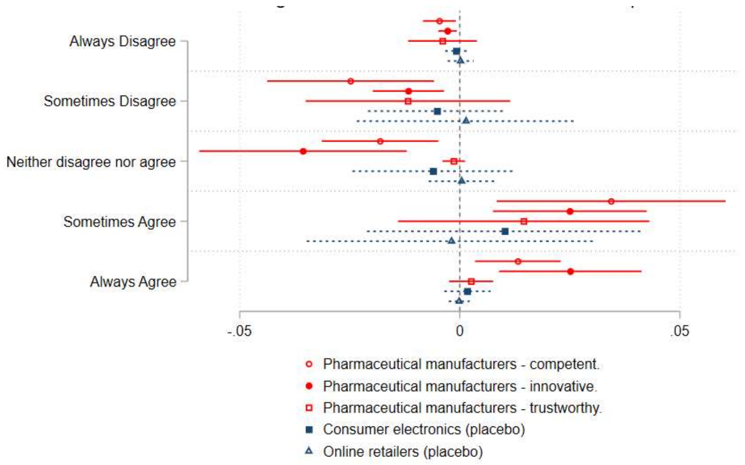


Notes: Figure 4a presents marginal effects estimated from ordered logit regressions of each outcome measure on treatment assignment, controlling for respondent age, sex, race, household income, and indicators for which of the inclusion criteria the respondent met (i.e., high cholesterol, current smoker, or overweight or obese). The ordered logit regressions estimated the effects of being randomized to either treatment group (i.e., either Treatment 1 or Treatment 2) compared to those who were randomized to the control group. Outcome measures represent consumer beliefs about pharmaceutical manufacturers and placebo industries, and include ordinal measures (ranging from 1 [Always disagree] to 5 [Always agree]) on perceptions of pharmaceutical manufacturers as being competent, innovative, and trustworthy, and placebo industries as being trustworthy.

### 4b-d: By Treatment Arm

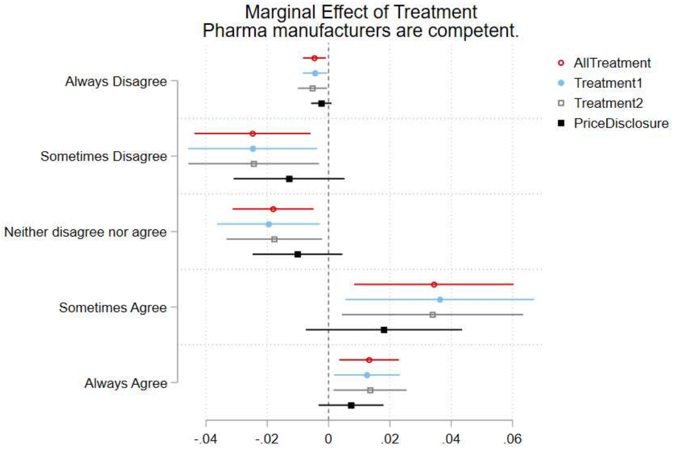


b)

© 2022 Eisenberg MD et al. *JAMA Health Forum.*

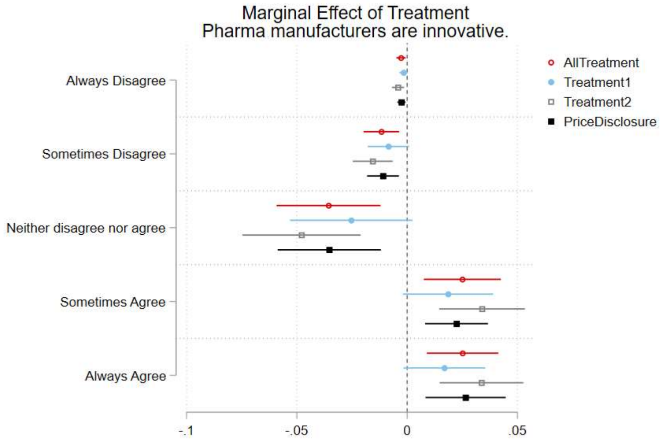


c)

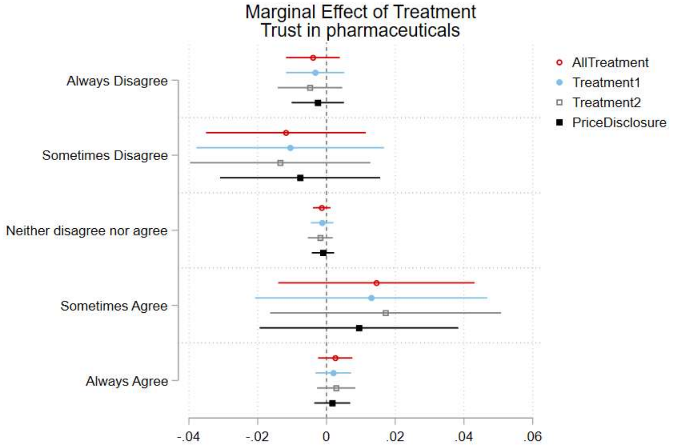


d)

Notes: eFigure 4b-d presents marginal effects estimated from ordered logit regressions of each outcome measure on treatment assignment, controlling for respondent age, sex, race, household income, and indicators for which of the inclusion criteria the respondent met (i.e., high cholesterol, current smoker, or overweight or obese). The coefficients labeled “All Treatment” represent results from ordered logit regressions that estimated the effects of being randomized to either treatment group (i.e., either Treatment 1 or Treatment 2) compared to those who were randomized to the control group). The coefficients labeled “Treatment 1” represent results from ordered logit regressions that estimated the effects of being randomized to receive Treatment 1 (DTCA without price disclosure) compared to those who were randomized to the control group. The coefficients labeled “Treatment 2” represent results from ordered logit regressions that estimated the effects of being randomized to receive Treatment 2 (DTCA with price disclosure) compared to those who were randomized to the control group. The coefficients labeled “Price Disclosure” compare the coefficients obtained from “Treatment 1” and “Treatment 2” regressions to present the differential effect of price disclosure on outcomes of interest. Outcome measures represent consumer beliefs about pharmaceutical manufacturers, and include ordinal measures (ranging from 1 [Always disagree] to 5 [Always agree]) on perceptions of pharmaceutical manufacturers as being competent, innovative, and trustworthy.

© 2022 Eisenberg MD et al. *JAMA Health Forum.*

## eFigure 5. Marginal Effect of Treatment on Health-Related Beliefs, By Treatment Arm

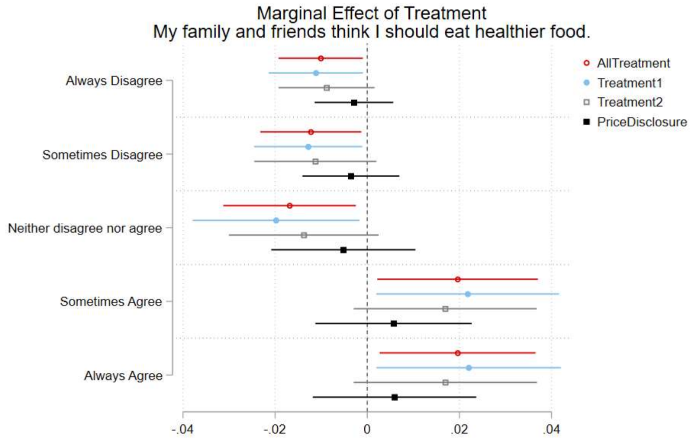


a)

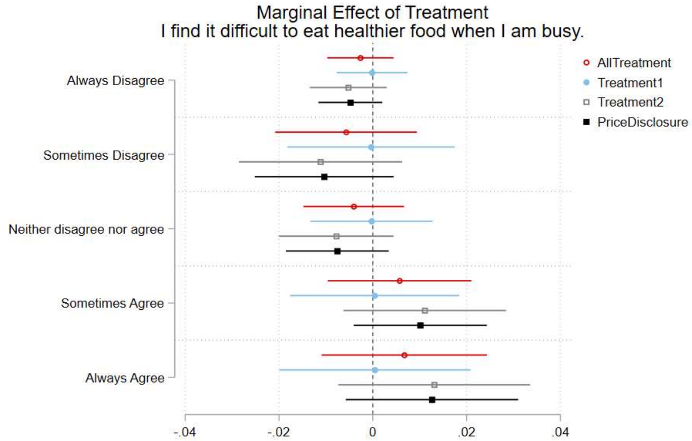


b)

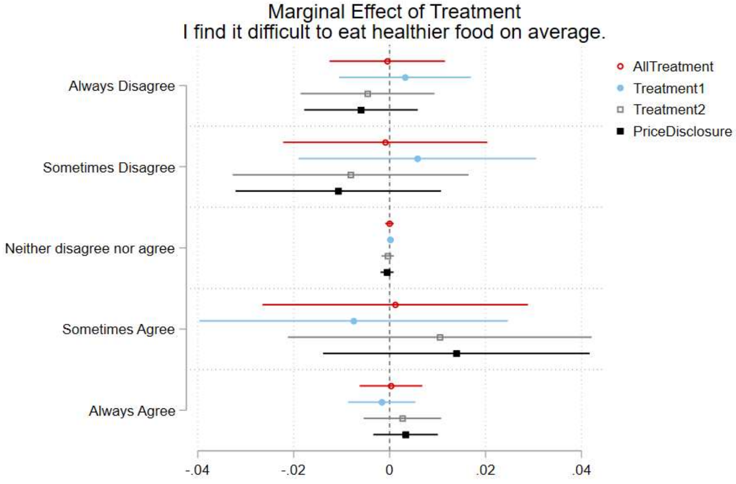


c)

© 2022 Eisenberg MD et al. *JAMA Health Forum.*

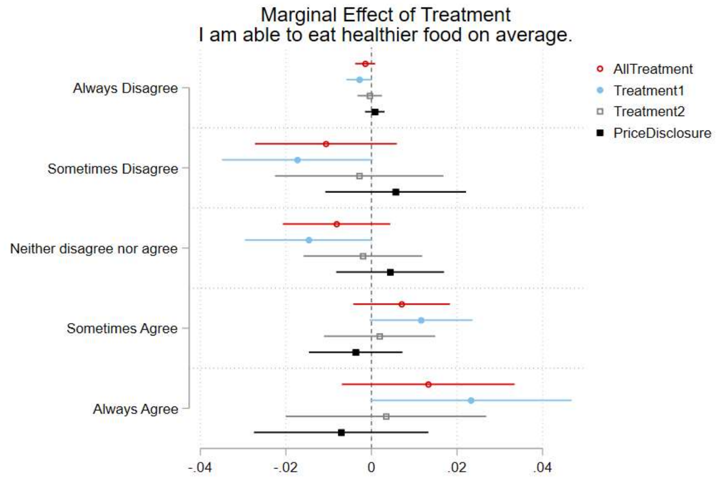


d)

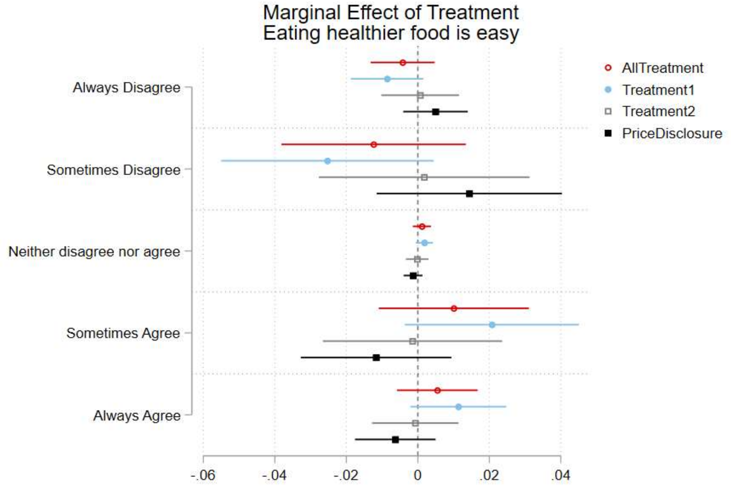


e)

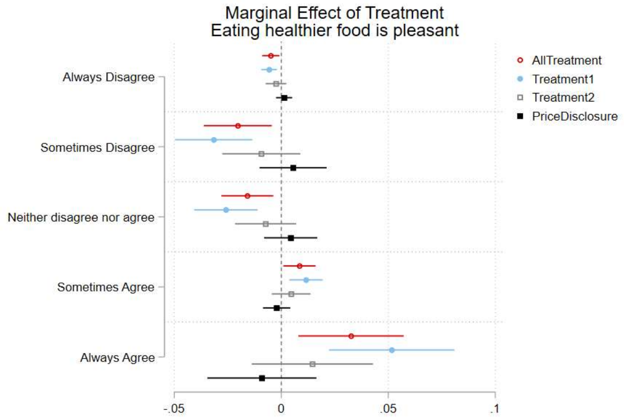


f)

© 2022 Eisenberg MD et al. *JAMA Health Forum.*

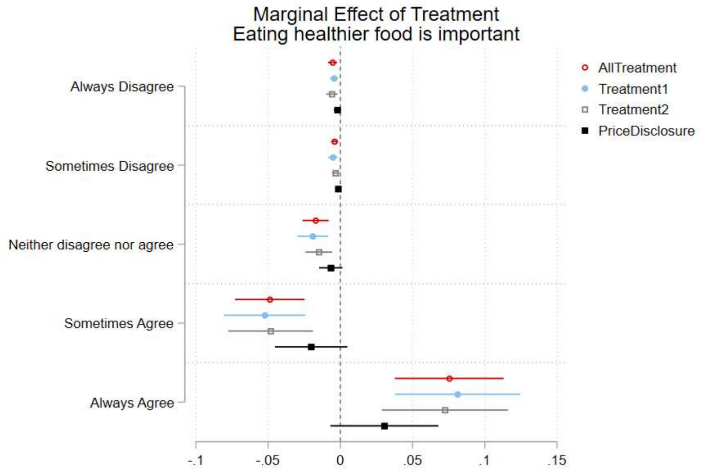


g)

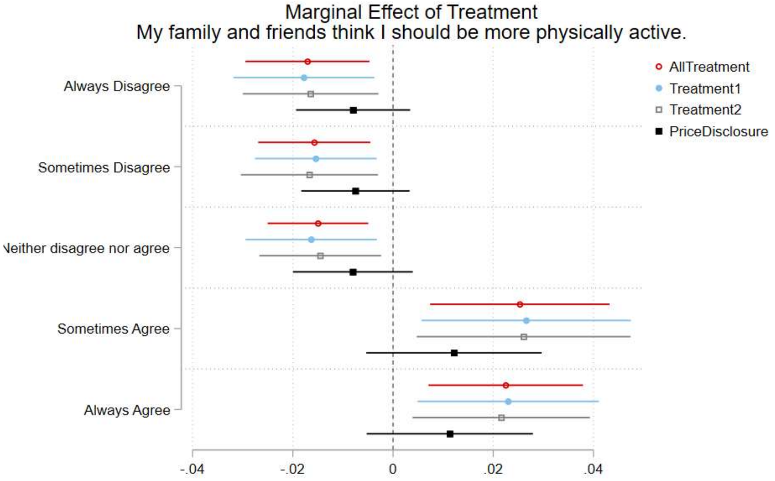


h)

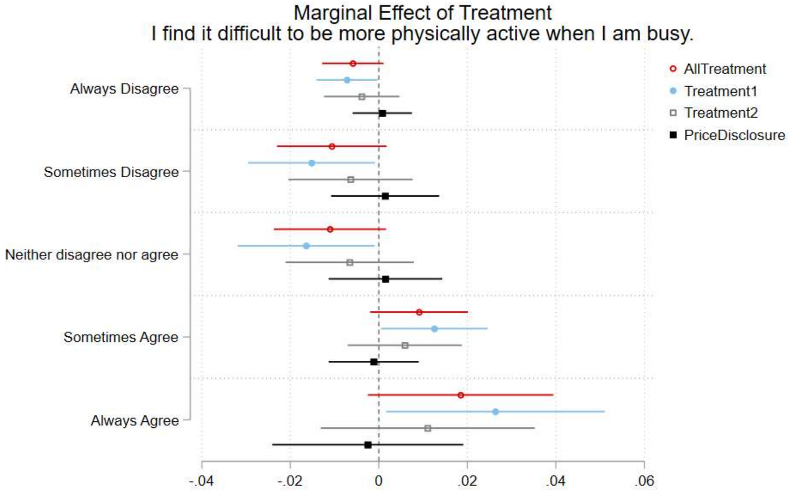


i)

© 2022 Eisenberg MD et al. *JAMA Health Forum.*

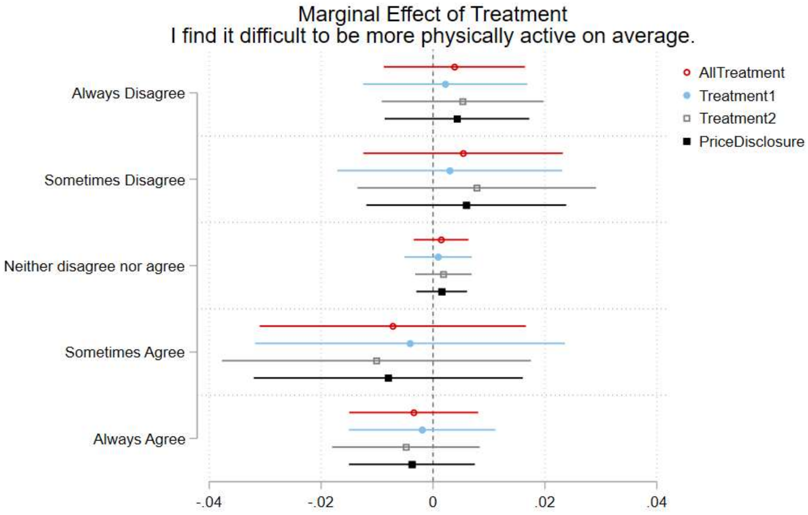


j)

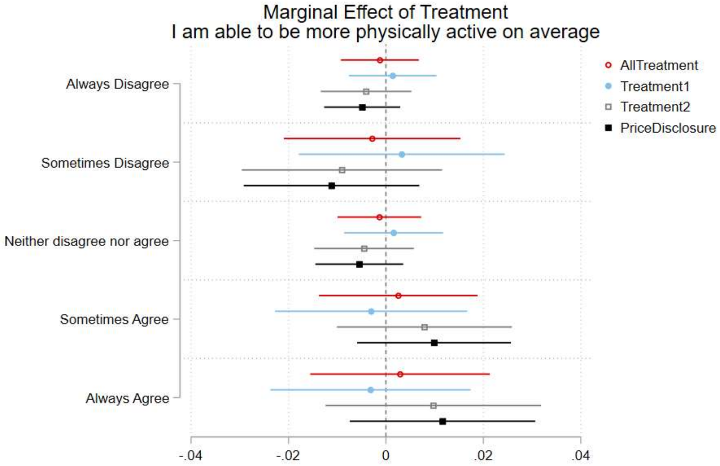


k)

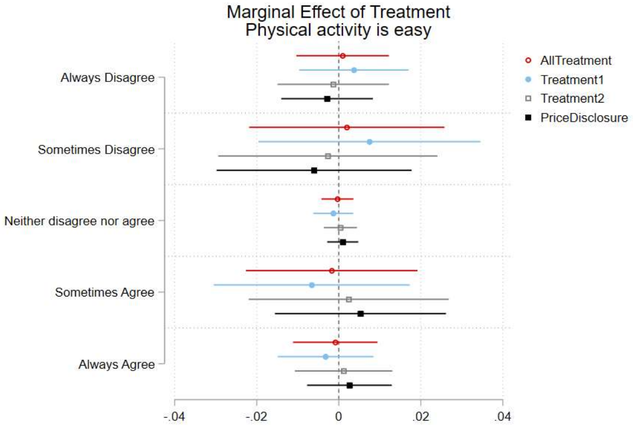


l)

© 2022 Eisenberg MD et al. *JAMA Health Forum.*

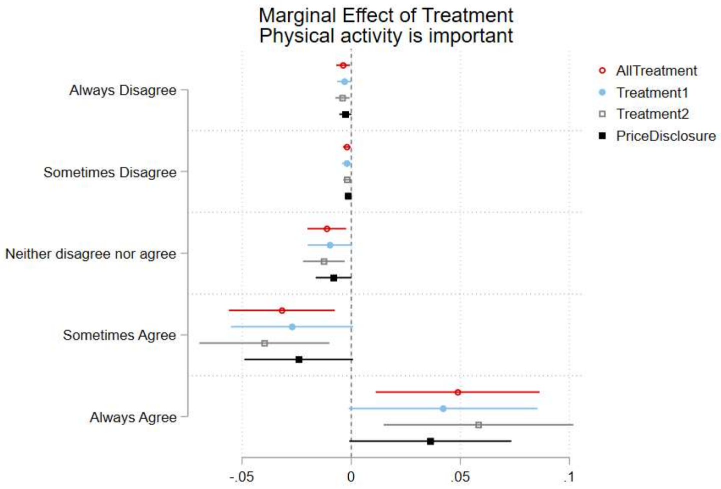


m)

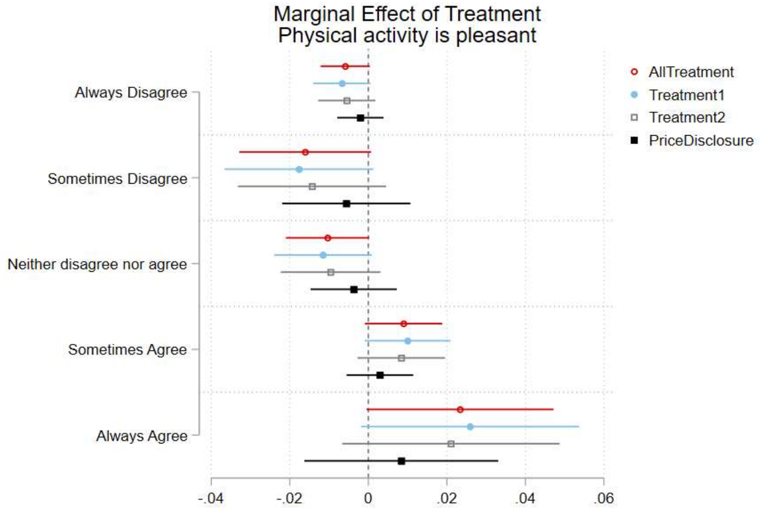


Notes: eFigure 5 presents marginal effects estimated from ordered logit regressions of each outcome measure on treatment assignment, controlling for respondent age, sex, race, household income, and indicators for which of the inclusion criteria the respondent met (i.e., high cholesterol, current smoker, or overweight or obese). The coefficients labeled “All Treatment” represent results from ordered logit regressions that estimated the effects of being randomized to either treatment group (i.e., either Treatment 1 or Treatment 2) compared to those who were randomized to the control group). The coefficients labeled “Treatment 1” represent results from ordered logit regressions that estimated the effects of being randomized to receive Treatment 1 (DTCA without price disclosure) compared to those who were randomized to the control group. The coefficients labeled “Treatment 2” represent results from ordered logit regressions that estimated the effects of being randomized to receive Treatment 2 (DTCA with price disclosure) compared to those who were randomized to the control group. The coefficients labeled “Price Disclosure” compare the coefficients obtained from “Treatment 1” and “Treatment 2” regressions to present the differential effect of price disclosure on outcomes of interest. Outcome measures represent perceived importance and difficulty of physical activity/dietary behavior. Perceived importance includes ordinal measures (ranging from 1[Always disagree] to 5[Always agree]) on beliefs related to physical activity and dietary behavior (e.g., “Eating healthier food is pleasant,” “Eating healthier food is important,” “Eating healthier food is easy”). Perceived difficulty includes ordinal measures (ranging from 1[Always disagree] to 5[Always agree]) on beliefs related to behavior-specific situations (e.g., “I am able to eat healthier food on average.,” “I find it difficult to eat healthier food on average,” “I find it difficult to eat healthier food when I am busy,” and “My family and friends think I should eat healthier food”).

**eFigure 6. Marginal Effect of Treatment on Medication-Related Intentions, Indirect Behavioral Intentions, Brand Perceptions, and Health-Related Beliefs (weighted regressions)**

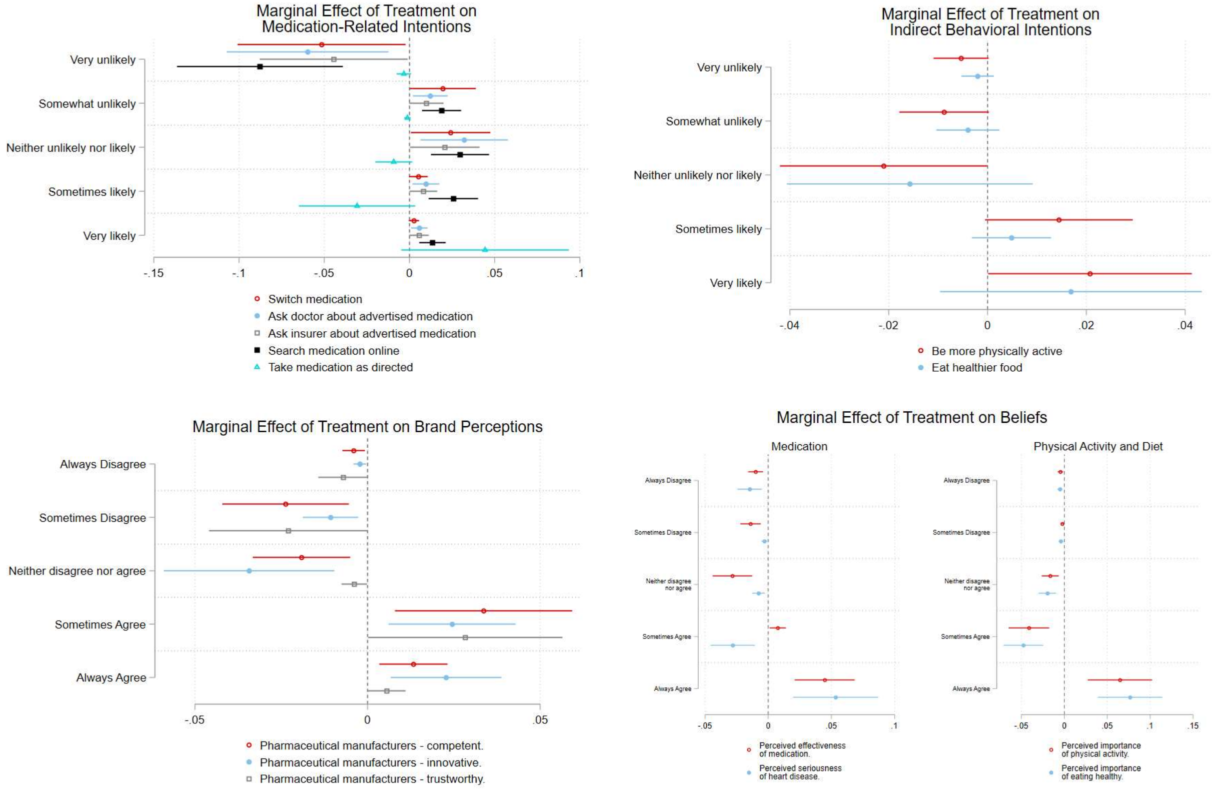


Notes: eFigure 6 presents marginal effects estimated from ordered logit regressions of each outcome measure on treatment assignment, controlling for respondent age, sex, race, household income, and indicators for which of the inclusion criteria the respondent met (i.e., high cholesterol, current smoker, or overweight or obese). All regressions are weighted by survey weights using geodemographic benchmarks from the U.S. Census Bureau's Current Population Survey (CPS).

© 2022 Eisenberg MD et al. *JAMA Health Forum.*

## eFigure 7. Marginal Effect of Treatment on Medication-Related Intentions, Indirect Behavioral Intentions, Brand Perceptions, and Health-Related Beliefs (without demographic controls)

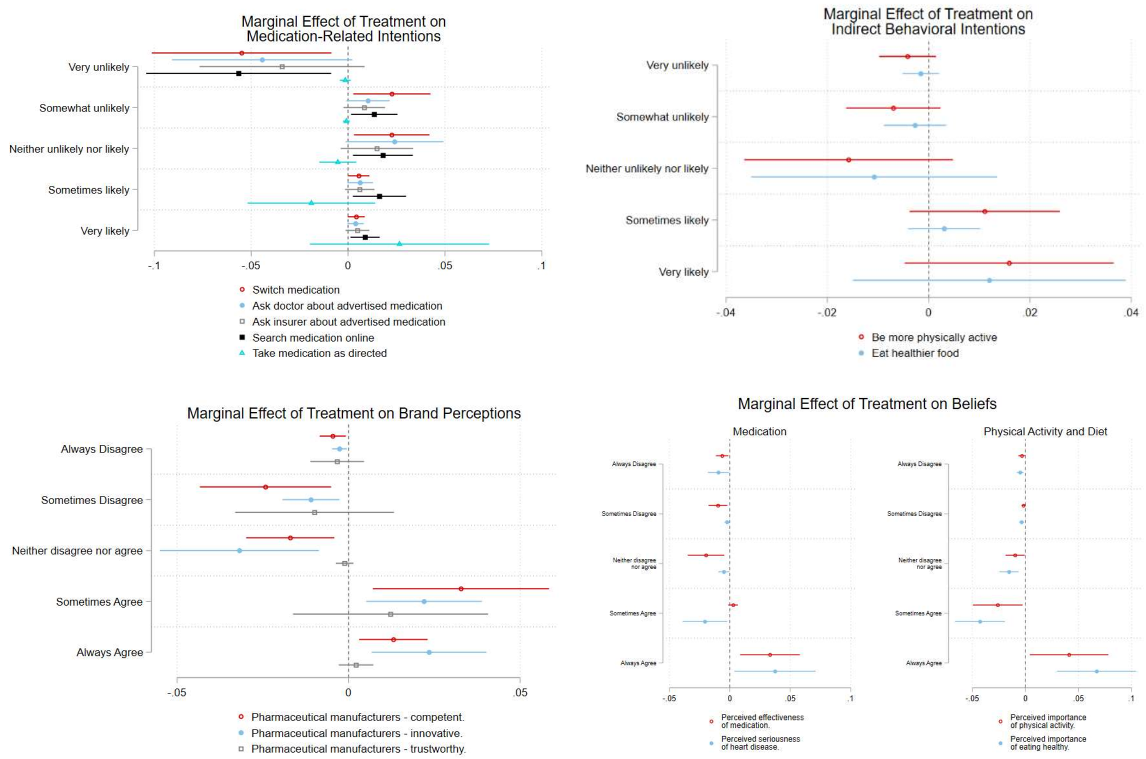


Notes: eFigure 7 presents marginal effects estimated from ordered logit regressions of each outcome measure on treatment assignment.

© 2022 Eisenberg MD et al. *JAMA Health Forum.*

**eTable 2. Descriptive Statistics for Medication-Related Intentions, By Treatment Arm**

| | Total | Control | Combined Treatment | p-value |
|---|---|---|---|---|
| | N=899 | N=292 | N=607 | |
| **Intend to take medication as directed** | | | | 0.082 |
| Never take as directed | 1% | 0% | 1% | |
| Rarely take as directed | 0% | 1% | 0% | |
| Sometimes take as directed | 2% | 3% | 1% | |
| Frequently take as directed | 9% | 9% | 8% | |
| Always take as directed | 88% | 87% | 89% | |
| **Intend to switch medication in next 2 weeks** | | | | 0.31 |
| Very unlikely | 85% | 89% | 83% | |
| Somewhat unlikely | 7% | 5% | 7% | |
| Neither unlikely nor likely | 6% | 4% | 6% | |
| Somewhat likely | 1% | 1% | 2% | |
| Very likely | 1% | 1% | 1% | |
| **Intend to ask doctor about Repatha** | | | | 0.023 |
| Very unlikely | 78% | 84% | 75% | |
| Somewhat unlikely | 5% | 2% | 6% | |
| Neither unlikely nor likely | 12% | 10% | 13% | |
| Somewhat likely | 3% | 1% | 4% | |
| Very likely | 3% | 3% | 3% | |
| **Intend to ask doctor about Brilinta** | | | | 0.11 |
| Very unlikely | 79% | 83% | 76% | |
| Somewhat unlikely | 4% | 2% | 5% | |
| Neither unlikely nor likely | 11% | 10% | 12% | |
| Somewhat likely | 2% | 1% | 2% | |
| Very likely | 3% | 3% | 3% | |
| **Intend to ask doctor about Entresto** | | | | 0.13 |
| Very unlikely | 78% | 83% | 76% | |
| Somewhat unlikely | 5% | 2% | 6% | |
| Neither unlikely nor likely | 11% | 10% | 12% | |
| Somewhat likely | 2% | 1% | 2% | |
| Very likely | 3% | 3% | 3% | |
| **Intend to ask doctor about Xarelto** | | | | 0.073 |
| Very unlikely | 78% | 83% | 75% | |
| Somewhat unlikely | 4% | 2% | 5% | |
| Neither unlikely nor likely | 12% | 10% | 13% | |
| Somewhat likely | 2% | 2% | 3% | |
| Very likely | 3% | 3% | 3% | |
| **Intend to ask insurance provider about medication** | | | | 0.13 |
| Very unlikely | 82% | 86% | 80% | |

© 2022 Eisenberg MD et al. *JAMA Health Forum.*

| | | | | |
|---|---|---|---|---|
| Somewhat unlikely | 5% | 3% | 6% | |
| Neither unlikely nor likely | 7% | 7% | 7% | |
| Somewhat likely | 3% | 2% | 3% | |
| Very likely | 3% | 3% | 2% | |
| **Intend to research medication online** | | | | 0.17 |
| Very unlikely | 73% | 78% | 71% | |
| Somewhat unlikely | 9% | 7% | 10% | |
| Neither unlikely nor likely | 8% | 6% | 8% | |
| Somewhat likely | 7% | 6% | 8% | |
| Very likely | 3% | 3% | 3% | |

Notes: eTable 2 reports responses to medication-related intentions for respondents randomized into either treatment arm (exposure to DTCA or exposure to DTCA with prices) compared to the control arm. P-value reports results from Chi-square tests.

© 2022 Eisenberg MD et al. *JAMA Health Forum.*

## eTable 3. Descriptive Statistics for Indirect Behavioral Intentions, By Treatment Arm

| | Total | Control | Combined Treatment | p-value |
|---|---|---|---|---|
| | N=2,874 | N=952 | N=1,922 | |
| **Intend to be more physically active** | | | | 0.75 |
| Always disagree | 4% | 4% | 4% | |
| Sometimes disagree | 8% | 8% | 7% | |
| Neither disagree nor agree | 36% | 37% | 36% | |
| Sometimes agree | 34% | 33% | 34% | |
| Always agree | 18% | 17% | 19% | |
| **Intend to eat healthier food** | | | | 0.31 |
| Always disagree | 3% | 3% | 2% | |
| Sometimes disagree | 5% | 6% | 4% | |
| Neither disagree nor agree | 32% | 31% | 33% | |
| Sometimes agree | 34% | 35% | 34% | |
| Always agree | 26% | 25% | 26% | |

Notes: eTable 3 reports responses to indirect behavioral intentions for respondents randomized into either treatment arm (exposure to DTCA or exposure to DTCA with prices) compared to the control arm. P-value reports results from Chi-square tests.

© 2022 Eisenberg MD et al. *JAMA Health Forum.*

## eTable 4. Descriptive Statistics for Brand Perceptions, By Treatment Arm

| | Total | Control | Combined Treatment | p-value |
|---|---|---|---|---|
| | N=2,874 | N=952 | N=1,922 | |
| **Pharmaceutical manufacturers are competent.** | | | | 0.043 |
| Always disagree | 3% | 3% | 2% | |
| Sometimes disagree | 17% | 17% | 16% | |
| Neither disagree nor agree | 31% | 33% | 30% | |
| Sometimes agree | 42% | 41% | 43% | |
| Always agree | 8% | 6% | 9% | |
| **Pharmaceutical manufacturers are innovative.** | | | | <0.001 |
| Always disagree | 1% | 1% | 1% | |
| Sometimes disagree | 6% | 7% | 5% | |
| Neither disagree nor agree | 27% | 27% | 26% | |
| Sometimes agree | 52% | 55% | 51% | |
| Always agree | 14% | 10% | 16% | |
| **Trust in pharmaceutical manufacturers** | | | | 0.041 |
| Always distrust | 6% | 6% | 6% | |
| Sometimes distrust | 27% | 26% | 27% | |
| Neither distrust nor trust | 27% | 30% | 26% | |
| Sometimes trust | 36% | 36% | 36% | |
| Always trust | 4% | 2% | 5% | |
| **Trust in consumer electronics businesses** | | | | 0.910 |
| Always distrust | 2% | 2% | 2% | |
| Sometimes distrust | 13% | 13% | 13% | |
| Neither distrust nor trust | 34% | 35% | 34% | |
| Sometimes trust | 47% | 46% | 47% | |
| Always trust | 4% | 4% | 4% | |
| **Trust in online retailers** | | | | 0.290 |
| Always distrust | 2% | 2% | 2% | |
| Sometimes distrust | 24% | 23% | 25% | |
| Neither distrust nor trust | 30% | 31% | 29% | |
| Sometimes trust | 41% | 42% | 41% | |
| Always trust | 2% | 1% | 2% | |

Notes: eTable 4 reports responses to brand perceptions for respondents randomized into either treatment arm (exposure to DTCA or exposure to DTCA with prices) compared to the control arm. P-value reports results from Chi-square tests.

© 2022 Eisenberg MD et al. *JAMA Health Forum.*

## eTable 5. Descriptive Statistics for Health-Related Beliefs, By Treatment Arm

| | Total | Control | Combined Treatment | p-value |
|---|---|---|---|---|
| | N=2,874 | N=952 | N=1,922 | |
| **How effective do you think medication is at treating heart disease?** | | | | 0.15 |
| Very ineffective | 3% | 4% | 3% | |
| Somewhat ineffective | 6% | 6% | 5% | |
| Neither ineffective nor effective | 15% | 16% | 14% | |
| Somewhat effective | 54% | 52% | 55% | |
| Very effective | 22% | 20% | 23% | |
| **How serious do you think heart disease is?** | | | | 0.081 |
| Very unserious | 5% | 5% | 5% | |
| Somewhat unserious | 1% | 1% | 1% | |
| Neither unserious nor serious | 3% | 3% | 3% | |
| Somewhat serious | 16% | 18% | 14% | |
| Very serious | 75% | 72% | 76% | |
| **Physical activity is important** | | | | 0.11 |
| Always disagree | 2% | 2% | 2% | |
| Sometimes disagree | 1% | 1% | 1% | |
| Neither disagree nor agree | 6% | 6% | 6% | |
| Sometimes agree | 29% | 33% | 27% | |
| Always agree | 61% | 58% | 63% | |
| **Eating healthier food is important** | | | | 0.010 |
| Always disagree | 2% | 2% | 2% | |
| Sometimes disagree | 1% | 2% | 1% | |
| Neither disagree nor agree | 6% | 7% | 6% | |
| Sometimes agree | 32% | 35% | 30% | |
| Always agree | 59% | 54% | 61% | |

Notes: eTable 5 reports responses to health-related beliefs for respondents randomized into either treatment arm (exposure to DTCA or exposure to DTCA with prices) compared to the control arm. P-value reports results from Chi-square tests.

© 2022 Eisenberg MD et al. *JAMA Health Forum.*

## eTable 6. Unadjusted and Romano-Wolf *P* values After Multiplicity Adjustment

| Category | Outcome | *P* value[a] | |
|---|---|---|---|
| | | Unadjusted | Romano-Wolf |
| Medication-related intentions | Intention to take medication as directed | .26 | .56 |
| Medication-related intentions | Intention to switch medication in next 2 wk | .05 | .18 |
| Medication-related intentions | Intention to ask physician about Repatha [Amgen] | .07 | .18 |
| Medication-related intentions | Intention to ask physician about Brilinta [AstraZeneca] | .14 | .55 |
| Medication-related intentions | Intention to ask physician about Entresto [Novartis] | .21 | .55 |
| Medication-related intentions | Intention to ask physician about Xarelto [Bayer] | .15 | .36 |
| Medication-related intentions | Intention to ask insurer about medication | .09 | .55 |
| Medication-related intentions | Intention to research medication online | .02 | .09 |
| Indirect behavioral intentions | Intention to be more physically active | .12 | .27 |
| Indirect behavioral intentions | Intention to eat healthier food | .26 | .36 |
| Brand perceptions | Pharmaceutical manufacturers are competent | .02 | .09 |
| Brand perceptions | Pharmaceutical manufacturers are innovative | .01 | .09 |
| Brand perceptions | Trust in pharmaceuticals | .26 | .64 |
| Brand perceptions | Trust in consumer electronics businesses | .47 | .64 |
| Brand perceptions | Trust in online retailers | .88 | .91 |
| Health-related beliefs | How effective do you think medication is at treating heart disease? | .01 | .09 |
| Health-related beliefs | How serious do you think heart disease is? | .02 | .27 |
| Health-related beliefs | Physical activity is pleasant | .11 | .55 |
| Health-related beliefs | Physical activity is important | .04 | .09 |
| Health-related beliefs | Physical activity is easy | .89 | >.99 |
| Health-related beliefs | I am able to be more physically active on average | .75 | >.99 |
| Health-related beliefs | I find it difficult to be more physically active on average | .67 | >.99 |
| Health-related beliefs | I find it difficult to be more physically active when I am busy | .06 | .09 |
| Health-related beliefs | My family and friends think I should be more physically active | .01 | .09 |
| Health-related beliefs | Eating healthier food is pleasant | .02 | .09 |
| Health-related beliefs | Eating healthier food is important | .01 | .09 |
| Health-related beliefs | Eating healthier food is easy | .35 | >.99 |
| Health-related beliefs | I am able to eat healthier food on average | .14 | .82 |
| Health-related beliefs | I find it difficult to eat healthier food on average | .94 | >.99 |
| Health-related beliefs | I find it difficult to eat healthier food when I am busy | .48 | >.99 |
| Health-related beliefs | My family and friends think I should eat healthier food | .03 | .27 |

[a]The unadjusted *P* value is obtained from ordinal logit regressions for each outcome. The Romano-Wolf *P* value represents Romano-Wolf stepdown-adjusted *P* values, which estimate the familywise error rate.

© 2022 Eisenberg MD et al. *JAMA Health Forum.*
